# The Fidelity of Dynamic Signaling by Noisy Biomolecular Networks

**DOI:** 10.1371/journal.pcbi.1002965

**Published:** 2013-03-28

**Authors:** Clive G. Bowsher, Margaritis Voliotis, Peter S. Swain

**Affiliations:** 1 School of Mathematics, University of Bristol, Bristol, United Kingdom; 2 Synthetic and Systems Biology, University of Edinburgh, Edinburgh, United Kingdom; North Carolina State University, United States of America

## Abstract

Cells live in changing, dynamic environments. To understand cellular decision-making, we must therefore understand how fluctuating inputs are processed by noisy biomolecular networks. Here we present a general methodology for analyzing the fidelity with which different statistics of a fluctuating input are represented, or encoded, in the output of a signaling system over time. We identify two orthogonal sources of error that corrupt perfect representation of the signal: dynamical error, which occurs when the network responds on average to other features of the input trajectory as well as to the signal of interest, and mechanistic error, which occurs because biochemical reactions comprising the signaling mechanism are stochastic. Trade-offs between these two errors can determine the system's fidelity. By developing mathematical approaches to derive dynamics conditional on input trajectories we can show, for example, that increased biochemical noise (mechanistic error) can improve fidelity and that both negative and positive feedback degrade fidelity, for standard models of genetic autoregulation. For a group of cells, the fidelity of the collective output exceeds that of an individual cell and negative feedback then typically becomes beneficial. We can also predict the dynamic signal for which a given system has highest fidelity and, conversely, how to modify the network design to maximize fidelity for a given dynamic signal. Our approach is general, has applications to both systems and synthetic biology, and will help underpin studies of cellular behavior in natural, dynamic environments.

## Author Summary

Cells do not live in constant conditions, but in environments that change over time. To adapt to their surroundings, cells must therefore sense fluctuating concentrations and ‘interpret’ the state of their environment to see whether, for example, a change in the pattern of gene expression is needed. This task is achieved via the noisy computations of biomolecular networks. But what levels of signaling fidelity can be achieved and how are dynamic signals encoded in the network's outputs? Here we present a general technique for analyzing such questions. We identify two sources of signaling error: dynamic error, which occurs when the network responds to features of the input other than the signal of interest; and mechanistic error, which arises because of the inevitable stochasticity of biochemical reactions. We show analytically that increased biochemical noise can sometimes improve fidelity and that, for genetic autoregulation, feedback can be deleterious. Our approach also allows us to predict the dynamic signal for which a given signaling network has highest fidelity and to design networks to maximize fidelity for a given signal. We thus propose a new way to analyze the flow of information in signaling networks, particularly for the dynamic environments expected in nature.

## Introduction

Cells are continuously challenged by extra- and intracellular fluctuations, or ‘noise’, [1]–[3]. We are only starting to unravel how fluctuating inputs and dynamic interactions with other stochastic, intracellular systems affect the behavior of biomolecular networks [4]–[9]. Such knowledge is, however, essential for studying the fidelity of signal transduction [10], [11] and therefore for understanding and controlling cellular decision-making [12]. Indeed, successful synthetic biology requires quantitative predictions of the effects of fluctuations at the single-cell level, both in static and dynamic environments [13]. Furthermore, sophisticated responses to signals that change over time are needed for therapeutics that involve targeted delivery of molecules by microbes [14], [15] or the reprogramming of immune cells [16]. Here we begin to address these challenges by developing a general framework for analysing the fidelity with which dynamic signals are represented by, or ‘encoded’ in, the output of noisy biomolecular networks.

## Results

### Two types of fidelity loss in dynamic signaling

**Figure 1:**
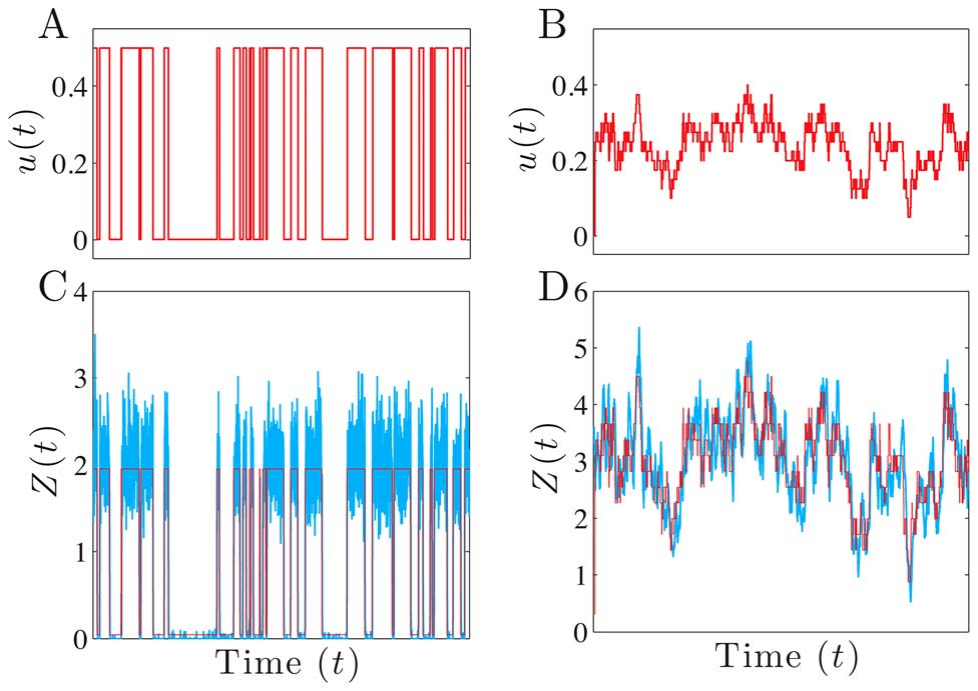
The dynamics of the protein output can result in a faithful representation of the current biological environment. We consider a 2-stage model of gene expression [22]. The extracellular environment or input, 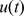, gives the current rate of transcription and the signal of interest 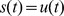. We model 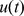 as either a 2-state Markov chain with equal switching rates between states (the states each have unconditional probability of 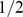) (A&C); or as proportional to a Poissonian birth-death process for a transcriptional activator (B&D; proportionality constant of 0.025). The transformed signals 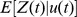 (in red, lower panels) are a perfect representation of 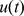, although protein levels 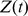 (in blue) are not. 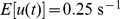, the lifetime 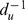 of 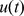 equals 1 hr, and the translation rate 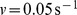. Degradation rates of mRNA and protein are chosen to maximize the fidelity, Eq. 7. The units for 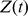 are chosen so that its variance equals one.

For cellular signaling to be effective, it should maintain sufficient fidelity. We wish to quantify the extent to which the current output of an intracellular biochemical network, 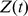, can represent a particular feature of a fluctuating input (Fig. 1). This *signal of interest*, 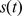, is generally a function of the history of the input, denoted 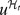. By its history, we mean the value of the input 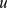 at time 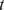 and at all previous times. The signal 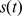 could be, for example, the level of the input at time 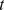 or a time average of the input over a time window in the most recent past. The output of the signaling network, 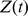, is able to perfectly represent the signal 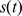 if 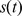 can be inferred exactly from 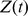 at all times, 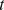. The system then has zero fidelity error. However, for a stochastic biochemical mechanism, a given value of 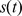 will map to multiple possible values of the output, 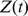.

We will assume that the conditional mean, 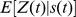, is an invertible function of 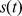: it takes different values for any two values of 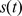. It is then a perfect representation of 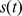. The output 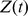 will, however, usually be different from 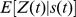 and have a fidelity error, defined as the difference between 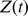 and 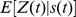. The notation 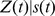 is read as 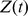 conditioned on, or given, the value of the variable 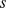 at time 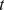. We use 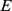, as for example in 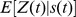, to denote averaging over all random variables except those given in the conditioning. Therefore 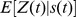 is itself a random variable: it is a function of the random variable 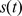 (we give a summary of the properties of conditional expectations in the SI).

Many response functions, 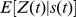, in biochemistry and physiology (for example, Hill functions) satisfy the requirement of invertibility or can be made to do so by defining 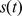 appropriately—for example, when a response exactly saturates for all input values above a threshold, those values can be grouped to form a single input state. Furthermore, we know from the properties of conditional expectations that 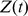 is closer to 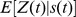 in terms of mean squared fidelity error than to any other representation (function) of 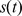 (SI).

**Figure 2:**
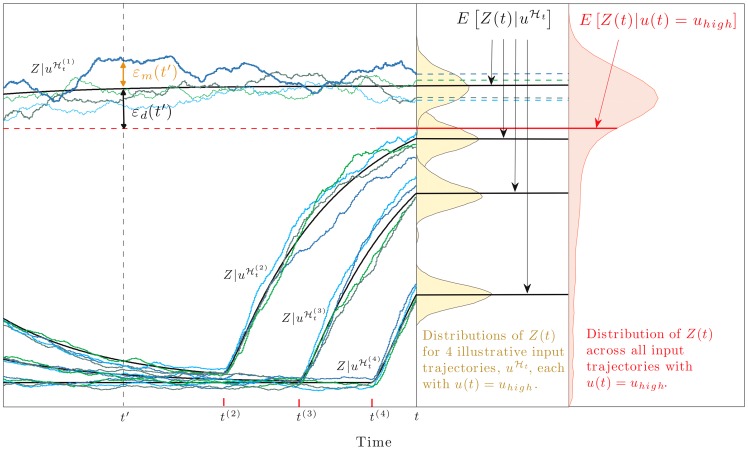
Dynamical error as the difference between two conditional expectations. To illustrate, we consider a 2-stage model of gene expression with the input, 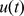, equal to the current rate of transcription, and the signal of interest 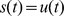. We model 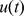 as a 2-state Markov chain and show simulated trajectories of the protein output, 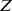, corresponding to four different input trajectories, 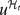. These input trajectories (or histories) all end at time 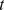 in the state 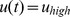 (not shown) and differ according to their times of entry into that state (labelled 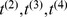 on the time axis; 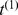 is off figure). 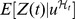 (black lines) is the average value of 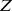 at time 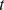 given a particular history of the input 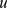: the random deviation of 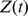 around this average is the mechanistic error 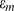 (shown at time 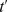 for the first realisation of 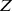). 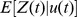 is the average or mean value of 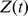 given that the trajectory of 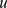 ends in the state 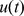 at time 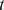. 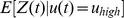 (red line) can be obtained by averaging the values of 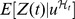 over all histories of 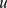 ending in 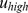. The mean is less than the mode of the distribution for 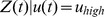 because of the distribution's long tail. 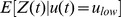, not shown, is obtained analogously. The dynamical error, 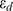, is the difference between 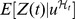 and 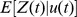 and is shown here for the first trajectory, 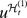. Fig. 3B shows data from an identical simulation model (all rate parameters here as detailed in Fig. 3B).

The difference between the conditional expectations 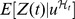 and, for example, 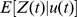 is important. The former, 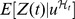, is the average value of the output at time 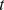 given a particular history of the input 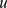. It will often coincide with the deterministic (macroscopic) solution when the same input trajectory is applied to the network. The output 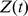 shows random variation around this average, 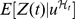, for identical realisations of the trajectory of 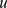. By contrast, 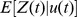 is the average value of 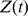 given that the trajectory of 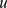 up to time 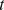 ends at the value 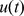. By the properties of conditional expectations, this is also the average value of 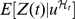 over all trajectories ending in the value 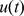: that is, 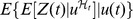 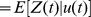. These mathematical definitions are illustrated diagrammatically in Fig. 2.

We distinguish between two types of error that reduce fidelity between 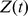 and 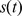.

#### *Dynamical error*

becomes significant when the response time of the signaling network is comparable to or longer than the timescale on which the signal of interest, 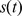, fluctuates. On average, the output 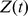 then responds to other features of the input history as well as to 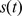. We define the dynamical error therefore as the difference between the average level of the output given a particular history of the input, 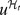, and the average level of the output given the signal of interest (a function of 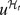):
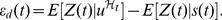
(1)The magnitude (variance) of the dynamical error is equal to 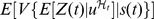, [7].

**Figure 3:**
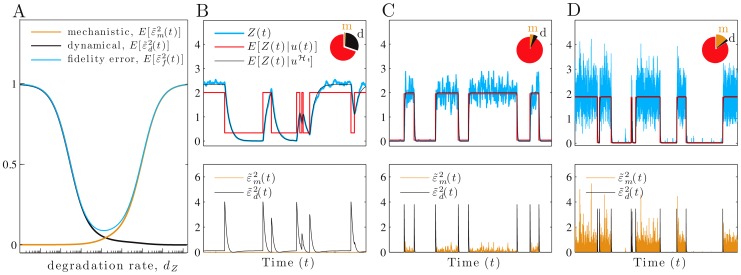
As the protein lifetime decreases, a trade-off between dynamical and mechanistic error determines fidelity. We consider a 2-stage model of gene expression with the input, 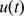, equal to the current rate of transcription, and the signal of interest 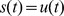. (A) The magnitude of the relative fidelity errors as a function of the protein degradation rate, 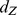 (from Eqs. 11, 12 and 13), using a logarithmic axis. (B–D) Simulated data with 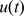 as in Fig. 1A. The units for 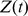 are chosen so that its variance equals one in each case (hence 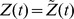 and 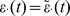). Pie charts show the fractions of the protein variance due to the mechanistic (m) and dynamical (d) errors and to the transformed signal. The latter equals 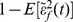. In B, the relative protein lifetime, 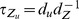, is higher than optimal (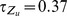) and fidelity is 2.2; in C, 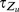 is optimal (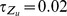) and fidelity is 10.1; and in D, 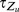 is lower than optimal (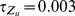) and fidelity is 5.3. Dynamical error, 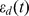, is the difference between 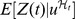 (black) and the faithfully transformed signal 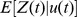 (red), and decreases from B to D, while mechanistic error increases. The lower row shows the magnitudes of the relative dynamical error (black) and relative mechanistic error (orange). All rate parameters are as in Fig. 1 A&C with 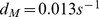, unless otherwise stated.

For example, if the signal of interest is the current value of the input, 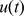, then 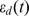 records a catch-up error if the network still ‘remembers’ (is still responding to) previous values of the input (Fig. 3). Since 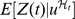 will generally be different for different input trajectories, it will generally differ from 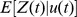 (which is an average over all input trajectories that end at 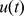, Fig. 2).

We can write the dynamical error as
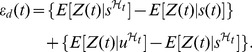
(2)


If fluctuations in 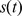 are slower than the response time of the system, then 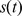 will be effectively constant over the ‘portion’ of its history detected by the output and the first term becomes zero because 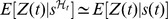. We note that the magnitude (variance) of 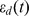 is always non-zero if the magnitude of this first term is non-zero because the two terms in Eq. 2 are uncorrelated (Methods). The second term quantifies the difference between the average effect on the output, 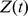, exerted by the history of the signal of interest and the average effect on the output exerted by the history of the input. This term would be non-zero, for example, if the input 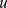 consists of multiple ligands that influence 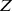, perhaps because of cross-talk between signaling pathways, but the signal of interest is only a function of the history of one of those ligands. This second term is zero, however, for the systems we will consider.

#### *Mechanistic error*

is generated by the inherent stochasticity of the biochemical reactions that comprise the signaling network. We define mechanistic error as the deviation of the current value of the output from its average value given a particular history of the input:
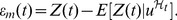
(3)

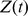
 departs from its average (given the realised input history) because of biochemical stochasticity (Fig. 2). The magnitude of mechanistic error is given by 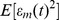, which equals 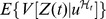.

Mechanistic error is related to intrinsic noise. Intrinsic variation measures the expected variation in 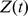 given the history of all the extrinsic variables [7], [8]. Extrinsic variables describe the influence of the rest of the cell and of the extracellular environment on, say, expression of a gene of interest [17] and would include, for example, levels of ATP and ribosomes as well as extracellular signals such as the input 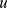. The magnitude of the mechanistic error measures, however, the expected variation in 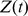 given the history of just one extrinsic variable, the input 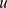. Mechanistic variation therefore also includes the effects of fluctuations in the levels of ATP and ribosomes on the signalling mechanism and is always greater than or equal to the intrinsic variation.

We then define the ***fidelity error***, 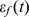, to be the sum of these two errors:
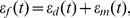
(4)which has zero mean, as do 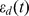 and 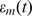. Fig. 1 shows fluctuating protein output levels, 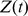, for a network that has high fidelity (small errors) for the signal of interest, there the current state of the environment, 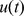.

### Orthogonal signal and error components

We can decompose the output 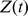 into the sum of the faithfully transformed or transmitted signal, 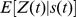, the dynamical error, and the mechanistic error:
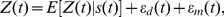
(5)for all times 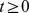. Eq. 5 is an orthogonal decomposition of the random variable 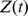
—each pair of random variables on the right-hand side has zero correlation (Methods). The variance of 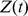 therefore satisfies
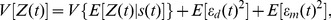
(6)where the magnitude of the fidelity error is given by 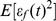, which is 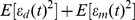 because of the orthogonality. This magnitude of the fidelity error is also equal to the expected conditional variance of the output, 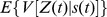. We note that we can generalize this decomposition, and thus extend our approach, for example, to study different components of the mechanistic error (Methods).

To compare signaling by different biochemical mechanisms, we normalize 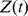 by the square root of its variance, writing 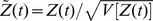, and define the fidelity as a signal-to-noise ratio:
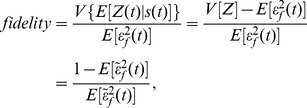
(7)for some signal of interest, 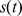. Eq. 7 is dimensionless and a montonically decreasing function of 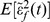. Indeed, we have shown that the maximal mutual information between 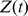 and 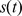 across all possible signal distributions is bounded below by a decreasing function of 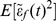 (and so an increasing function of our fidelity), for a suitable choice of distribution of the signal 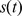 and when 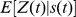 is an invertible function of 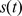 [7].

Comparing biochemical systems using the fidelity measure is equivalent to comparison based on the magnitude of the fidelity error, 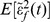, where 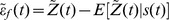 and the error is measured in units of the standard deviation of the output. Eq. 7 is maximized when 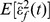 is minimized. One minus the magnitude of the fidelity error is the fraction of the variance in the output that is generated by the signal of interest. In information theoretic approaches, normalizing the output by its standard deviation is also important, because the normalization allows determination of the number of ‘unique’ levels of output that can be distinguished from one other despite the stochasticity of the output, as least for Gaussian fluctuations [18].

When 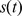 and 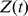 have a bivariate Gaussian distribution, the instantaneous mutual information, 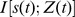, is monotonically related to the fidelity and exactly equal to 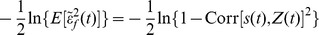 [7], where 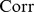 denotes the correlation coefficient. Also in this Gaussian case, 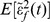 is equal to the minimum mean squared error (normalised by 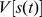) between 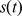 and the linear, optimal estimate, 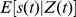. (This is the optimal ‘filter’ when only the current output 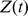 is available, although typically a filter such as the Wiener filter would employ the entire history of 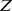 up to time 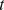.) Gaussian models of this sort for biochemical signalling motifs were considered in [19], with instantaneous mutual information expressed in terms of a signal-to-noise ratio equivalent (for their models) to the fidelity of Eq. 7. Such Gaussian models (if taken literally, rather than used to provide a lower bound on the information capacity [19]) would imply that the input-output relation, 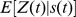, is linear and that 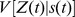 does not depend on 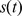 (by the properties of the multivariate normal distribution). Our approach requires neither assumption.

Whenever 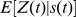 is a linear function of 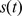, that is 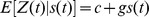 for constants 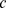 and 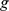, we consider 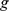 to be the gain for the signal of interest 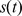 [19]. The fidelity then depends on the ratio of the squared gain to the fidelity error and is given by 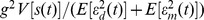.

#### The dynamic signal with maximum fidelity for a given input process

Suppose that the input process 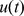 is given and we want to choose from among all functions or statistics of the input history that ‘signal of interest’, 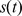, for which the network achieves the highest fidelity. An immediate implication of Eq. 7 is that it identifies the signal of interest with the highest fidelity. Since 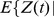 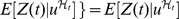, the dynamical error is zero when
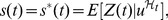
(8)from Eq. 1. This choice of 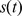 therefore maximizes fidelity for all signaling networks: it minimizes the magnitude of the fidelity error (Eq. 6), because 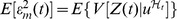 and 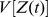 do not depend on 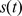. The variance of 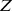 only changes with the biochemistry of the network and the input process. We will give an example of such a signal of interest that maximizes fidelity in Eq. 9.

### Analyzing networks with fluctuating inputs

Methods of analysis of stochastic systems with dynamic inputs are still being developed. We argue that deriving expectations of network components conditional upon the histories of stochastic inputs is a powerful approach. We have developed three methods to determine components of Eqs. 5 and 6 (SI):

- An exact analytical method, applicable to linear cascades and feedforward loops, based on the observation that moments calculated from a chemical master equation with propensities that are the appropriate functions of time are conditional moments, where the conditioning is on the history of the inputs at time 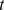 and on the initial conditions.
- A Langevin method that can include non-linearities, requires stationary dynamics, and whose accuracy as an approximation improves as typical numbers of molecules grow.
- A numerical method, applicable to arbitrary biomolecular networks and signals of interest—based on a modification of the Gillespie algorithm allowing time-varying, stochastic propensities—that uses a ‘conjugate’ reporter to estimate the mechanistic error [7] and a simulated sample from the distribution of the signal-output pair, 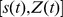, to estimate the conditional means, 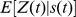.

We note that our methods require that the inputs can be modeled as exogenous processes that are unaffected by interactions with the biochemistry of the signaling network (a distinction emphasised in [20]). By an exogenous process we mean one whose future trajectory is independent, given its own history, of the history of the biochemical system. This model for an input is reasonable, for example, when the input is the level of a regulatory molecule, such as a transcription factor, that has relatively few binding sites in the cell.

### Analyzing signal representation by gene expression

Transcriptional regulation is a primary means by which cells alter gene expression in response to signals [21]. We now provide an exact, in-depth analysis of a two-stage model of gene expression [22] where the fluctuating input, 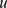, is the rate (or propensity) of transcription and the signal of interest, 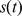, equals the current value of the input, 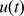. For example, 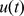 may be proportional to the extracellular level of a nutrient or the cytosolic level of a hormone regulating a nuclear hormone receptor.

The cellular response should account for not only the current biological state of 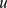 but also future fluctuations. If we consider an input that is a Markov process, future fluctuations depend solely on the current value 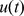, and the cell would need only to ‘track’ the current state as effectively as possible and then use the representation in protein levels to control downstream effectors. These ideas are related to those underlying predictive information [23], [24].

Our analysis requires only the stationary mean and variance of the input 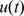 and that 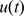 has exponentially declining ‘memory’ (SI). Consequently, the autocorrelation function of 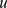 is a single exponential with autocorrelation time 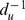 (the lifetime of fluctuations in 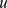). Examples include a birth-death process or a two-state Markov chain. We can generalize using, for example, weighted sums of exponentials to flexibly model the autocorrelation function of 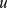.

Solving the ‘conditional’ master equation with a time-varying rate of transcription, we find that the conditionally expected protein level is a double weighted ‘sum’ of past levels of the signal 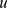 (SI):
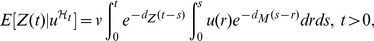
(9)(where for simplicity the equation is stated for the case of zero initial mRNA and protein). We denote the rate of translation per molecule of mRNA by 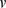, the rate of mRNA degradation per molecule by 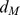, and the rate of degradation of protein per molecule by 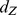. The most recent history of the input 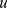 exerts the greatest impact on the current expected output, with the memory of protein levels for the history of the input determined by the lifetimes of mRNA and protein molecules. Eq. 9 gives the signal of interest, 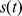 (a function of the history of the fluctuating transcription rate), that gene expression transmits with the highest fidelity to protein levels (see Eq. 8). Notice that the current value of the input, 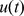, cannot be recovered exactly from 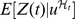, which is therefore not a perfect representation of 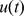.

We find, by contrast, that 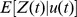 is an invertible, linear function of 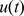:
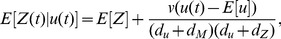
(10)when the dynamics reach stationarity, and that the stationary unconditional mean is 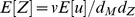 (SI). Notice that 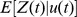 does not converge for large 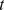 to the average ‘steady-state’ solution for a static 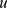, but depends on 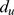. The discrepancy between Eqs. 9 and 10 results in dynamical error with non-zero magnitude (Fig. 3B).

Using our solutions for the conditional moments, we can calculate the variance components of Eq. 6 (SI). For the faithfully transformed signal, when 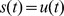, we have
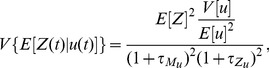
(11)where 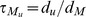 is the ratio of the lifetime of mRNA to the lifetime of fluctuations in 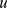, and 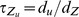 is the ratio of the lifetime of protein to the lifetime of fluctuations in 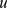. The magnitude of the dynamical error is in this case proportional to Eq. 11

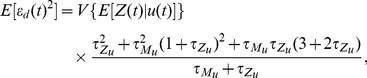
(12)and the magnitude of the mechanistic error satisfies
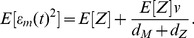
(13)When the autocorrelation time of 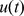 becomes large (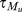 and 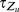 tending to zero), the dynamical error 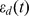 therefore vanishes (Eq. 12). In this limit, the output effectively experiences a constant input 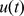 during the time ‘remembered’ by the system.

To gain intuition about the the effect of relative lifetimes on the fidelity of signaling, we first suppose the mechanistic error is small relative to 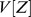. Eq. 7 then becomes simply 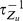 if protein lifetime is large relative to mRNA lifetime, 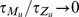 (as expected for many genes in budding yeast [25]). The fidelity thus improves as the protein lifetime decreases relative to the lifetime of fluctuations in 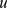, and the output is able to follow more short-lived fluctuations in the signal. This observation is only true, however, for negligible mechanistic error.

### Tradeoffs between errors can determine signaling fidelity

It is the aggregate behavior of dynamical and mechanistic errors as a fraction of the total variance of the output that determines signaling fidelity, Eq. 7. Effective network designs must sometimes balance trade-offs between the two types of error.

#### Increasing biochemical noise can enhance signaling fidelity

Predicting changes in fidelity requires predicting whether changes in the magnitude of the dynamical error relative to 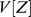, denoted 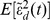, either dominate or are dominated by changes in the magnitude of the mechanistic error relative to 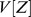, denoted 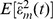. For example, shorter protein lifetimes can decrease the absolute value of both the dynamical error and the mechanistic error (the output has a lower mean—Eq. 13). We calculated for all parameter space the sensitivities of the magnitude of the two (relative) errors with respect to changes in the protein lifetime, 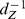 (using Eqs. 11, 12, and 13). We found that although the relative magnitude of the dynamical error decreases with shorter protein lifetime, the relative magnitude of the mechanistic error increases. The sign of the overall effect on the relative fidelity error can therefore be positive or negative (Fig. 3A), and consequently fidelity is maximized by a particular protein lifetime, 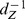 (Fig. 3B–D).

Similar trade-offs have been observed before in signal transduction. For example, tuning the protein's degradation rate can also maximize the instantaneous mutual information, at least for Gaussian models [19]. As the protein degradation rate increases, although the fidelity error 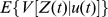 decreases, there is a trade-off because the gain also decreases. In our model the gain, 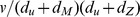 (Eq. 10), is decreasing in 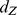 and we observe the same tradeoff.

Further, the trade-off between the two relative errors has some similarities with trade-offs that occur with Wiener filtering [26]. There, however, the entire output history is used to optimally estimate (or reconstruct) the signal of interest. In contrast, we consider representation of 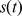 only by the current output 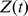.

The rule-of-thumb that increasing stochasticity or noise in signaling mechanisms reduces signaling fidelity is broken in this example. Such statements typically ignore the effect of dynamical error, but here reductions in relative dynamical error can more than compensate for gains in relative mechanistic error. Both errors should be included in the analysis.

#### Feedback can harm signaling fidelity

Intuitively we might expect that feedback can improve signaling fidelity because feedback affects response times. For example, autoregulation affects the mean time to initiate transcription: it is reduced by negative autoregulation [27] and increased by positive autoregulation [28]. We introduce autoregulation into our model of gene expression, interpreting again 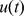 as proportional to the fluctuating level of a transcriptional activator and allowing the protein 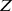 to bind to its own promoter. For negative feedback, the rate of transcription becomes 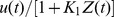; for positive feedback, it becomes 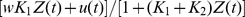, with 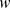 the rate of transcription from the active promoter (SI). We impose 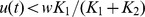 so that the transcription rate increases with 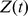 for a given 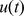. Increasing 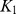 increases the strength of the feedback in both cases. We note that other models of autoregulation may give different conclusions, and that the transcription rate depends linearly on 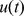 in our models.

We let the signal of interest 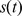 again be 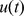. To proceed we calculate the sensitivities of the magnitudes of the fidelity errors using our Langevin method with the input an Ornstein-Uhlenbeck process. We determine their signs with respect to changes in feedback strength by randomly sampling a biophysically plausible parameter space (SI). As we sample, the parameter space governing fluctuations of 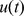 is also explored. We find excellent agreement between our Langevin and numerical, simulation-based approach (SI). Since we calculate sensitivities, we are examining the effect of changing feedback strength, 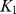, while holding other network parameters constant. This process both imitates the incremental change often expected during evolution and the way that network properties tend to be manipulated experimentally. When comparing the fidelity error of the signal representations for different 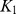 using Eq. 7, we implicitly normalise the variance of the output to one in order to ensure fair comparison.

Consider first the static case where the fluctuations in 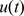 are sufficiently slow relative to the timescales of the transduction mechanism that the input is effectively constant (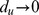 with fixed 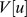). As expected (Eq. 1), 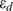 converges to zero as 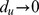. With a static input, negative autoregulation is expected to reduce the variances of the response, 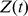, for each value of the input [29]. The mechanistic variance is therefore expected to decrease, and does so in all models sampled as 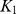 increases. We can show analytically (SI) that the suppression of mean levels also decreases the variance of the conditional mean, the ‘signal’ variance 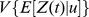, and so the total variance of the output decreases. We find that the decrease in mechanistic variance cannot outweigh the decreased signal variance, and the fidelity always decreases with increasing feedback (increasing 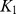). Such a reduction in information transfer through negative feedback has recently been observed experimentally [10]. For positive autoregulation, the mechanistic variance increases with 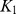, which dominates any increase in the signal variance observed at low values of 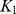. Relative mechanistic error again rises and fidelity therefore decreases.

For a static 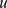, therefore, neither negative nor positive autoregulation improves signaling fidelity. As the strength of feedback becomes large, the transcriptional propensity tends to zero for negative feedback and to the constant 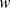 for positive feedback (with fixed positive 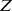), and the propensities for different 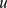 become indistinguishable as functions of 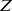 (SI). Signaling is correspondingly compromised in both cases.

**Figure 4:**
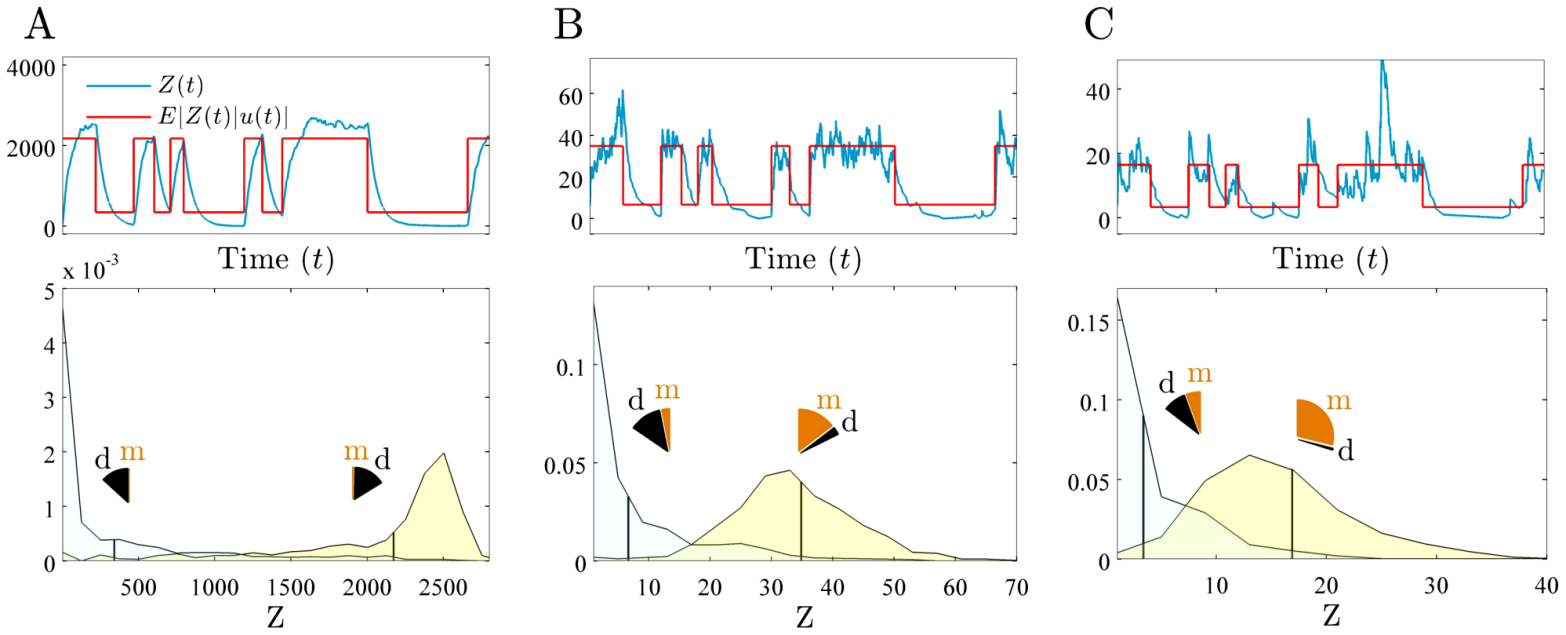
Increasing the strength of negative feedback decreases fidelity. We consider a 2-stage model of gene expression with the signal of interest 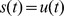, and with 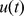 proportional to the level of a transcriptional activator. We simulate 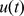 as in Fig. 1A. Upper row compares the time course of the protein output (blue) to the faithfully transformed signal (red), 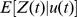. Lower row shows the distributions for the output, 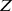, that correspond to each of the two possible values of the input, 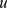 (low and high). Vertical lines indicate the means of the distributions. Pie charts show the fractions of the variance of each (conditional) distribution due to dynamical (d) and mechanistic (m) error, weighted by the probability of the input state: summing these gives the overall magnitude (variance) of the dynamical and mechanistic errors. (A) No feedback (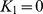), fidelity equals 2.4. (B) Intermediate feedback (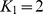), fidelity equals 2.0. (C) Strong feedback (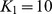), fidelity equals 1.3. As the strength of feedback increases, the underlying state of the input is more difficult to infer (the conditional distributions overlap more) because increasing (relative) mechanistic error dominates the decreasing (relative) dynamical error. Note the decrease in the (relative) dynamical error when 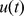 is in its high state (yellow conditional distribution) because stronger negative feedback gives faster initiation of transcription. Transcription propensities are given by 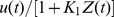, and all parameters except 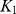 are as in Fig. 3B.

These findings essentially still hold when the input is dynamic. For negative autoregulation, all three components of the output variance decrease with 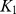. The relative dynamical error decreases with 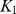, but this decrease is typically outweighed by an increase in the relative mechanistic error, and the overall fidelity deteriorates (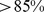 of cases sampled and Fig. 4). Any reduction in fidelity error, 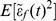, was negligible (the difference from the fidelity error when 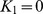 was always less than 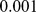). We note that this conclusion is in contradistinction to the finding (using a linear Gaussian model) that negative feedback does not affect information transfer between entire input and output trajectories [30]. For positive feedback, both the mechanistic variance and the relative mechanistic error increase with 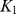 (for all models sampled). This mechanistic effect dominates the relative dynamical error, which can change non-monotonically with 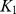, and fidelity again deteriorates.

Our results are consistent with the intuition that, although negative feedback reduces the absolute mechanistic error (fewer molecules) and absolute dynamical error (faster response times), negative feedback also decreases the dynamic range of the output. The fidelity therefore does not improve because the output distributions corresponding to each value of 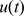, despite being tighter, are also located closer together (Fig. 4). Positive feedback acts in the opposite way, with increasing variance in the (conditional) output distributions overwhelming any increase in the dynamic range of the output.

To explore what happens when the effect of feedback on the dynamic range is directly controlled, we investigated the effect of varying 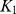 in our negative feedback model while simultaneously altering the translation rate (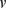) to hold the system's ‘gain’ constant (SI). In our model, the faithfully transformed signal is a linear function of 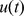: 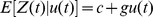, where 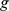 is the gain. If only 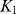 is varied and the translation rate kept fixed, then the gain is always less than the gain when 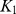 is zero. The signal variance or ‘dynamic range’, 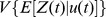, is equal to 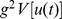, which is also therefore held constant as we vary 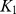 at constant gain. The fidelity is 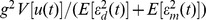.

For static signals, we again find the fidelity almost always decreases with increasing negative feedback strength, 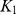: the absolute mechanistic error now increases with increasing 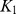, presumably because of the decreased rate of translation. For dynamic signals we find, for the vast majority of cases, an optimal feedback strength, 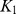, above and below which fidelity deteriorates. With increased 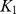, although the absolute mechanistic error increases, the absolute dynamical error decreases, when we compare randomised initial parameterisations with the 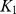 that maximises fidelity. When 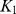 decreases compared to its initial value, these errors have the opposite behavior. At constant gain, the tradeoff between dynamical and mechanistic error is thus still observed, as is the harmful effect of too strong a negative feedback.

#### Combining outputs from multiple cells improves fidelity

When a physiological response corresponds to the average output of multiple cells, the magnitude of the mechanistic error is that for a single cell divided by the number of cells in the group (for identical and independent cells receiving the same input). This reduction arises because the magnitude of the mechanistic error is now the variance of the average mechanistic error of the cells in the group. The dynamical error, Eq. 1, however, is the same as the dynamical error of each individual cell: expectations of the average response equal the expectations of the response of each single cell when the cells are identical. Therefore the fidelity for any signal of interest, 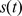, increases if the average or aggregate output of a group of cells is used (SI). Measuring the collective response of small groups of cells, Cheong *et al.* indeed found that information capacity increased significantly compared to that of a single cell [10], and averaging of individual cellular responses is believed to increase the precision of gene expression during embryonic development [31].

**Figure 5:**
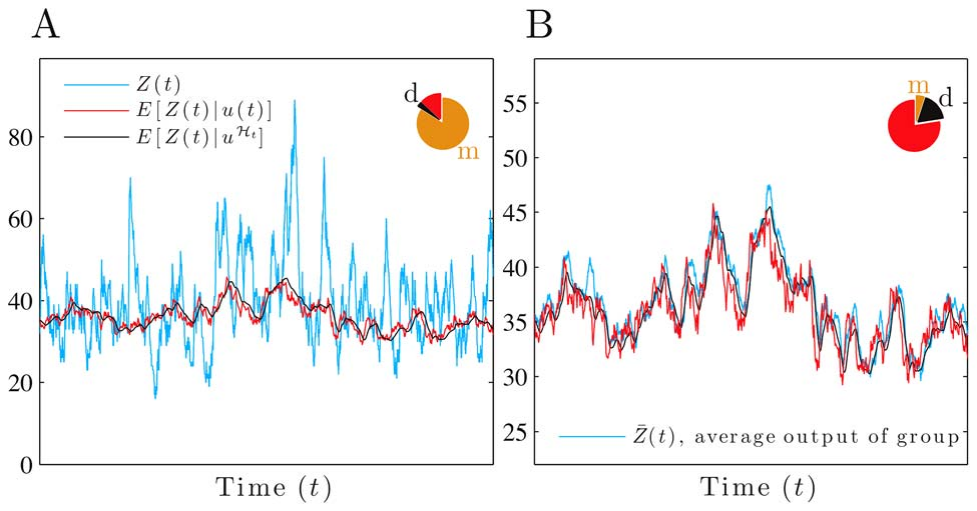
The fidelity of the collective response of a group of cells exceeds that of a single cell. We consider a 2-stage model of gene expression with the signal of interest 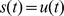, and with 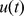 proportional to the level of a transcriptional activator and modeled as an Ornstein-Uhlenbeck process. The unconditional distribution of 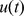 is therefore Gaussian. Pie charts show fractions of the protein variance due to the mechanistic (m) and dynamical (d) errors and are computed using our Langevin method (SI). (A) For a single cell with negative autoregulation (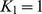), fidelity is low and equal to 0.2, with a dominant mechanistic error. (B) For 100 identical and independent cells (given the input's history), with negative autoregulation (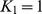): fidelity between 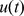 and the average protein output for the group is higher and equal to 3.5. All parameters as in Fig. 3B except 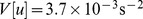.

Although negative feedback reduces relative dynamical error, it increases relative mechanistic error in individual cells. At the level of the collective response of multiple cells, the deleterious effect on mechanistic error is attentuated (Fig. 5). Using a population of 100 independent and identical cells we find that adding negative feedback now improves fidelity in the majority of cases, with moderate reductions in (relative) fidelity error (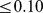) for our parameter space. Adding positive feedback never significantly improves overall fidelity (all observed reductions 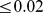). Furthermore, negative feedback can often significantly reduce the number of cells needed to achieve the same fidelity as, say, 100 cells that lack feedback (less than 10 cells are needed 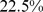 of the time and less than 50 cells 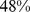 of the time when sampling from our parameter space).

### Designing dynamic networks in synthetic biology

Our framework naturally adapts to the scenario of controlling a network output to approach a desired ‘target’ response when, for example, the cell's environment changes. Combined with model search procedures for synthetic design [32], it is a promising approach to the design of synthetic biomolecular networks. If the target response is given by 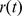, which is a function of the input history, then to guide the design process, we can decompose the error 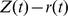 analogously to Eq. 5 and find an equivalent to Eq. 6, a dissection of the network performance into orthogonal components (SI).

## Discussion

Cells use the information conveyed by signaling networks to regulate their behavior and make decisions. Not all features of the input trajectory will, however, be relevant for a particular decision, and we define the fidelity between the output of the network and a signal of interest, 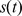, which is a function of the input trajectory. Information encoded in upstream fluctuations must eventually either be lost or encoded in current levels of cellular constituents. We have therefore focused on the fidelity with which 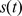 is represented by the current output, 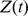.

Using an orthogonal decomposition of the network's output into the faithfully transformed signal and error terms, we are able to identify two sources of error – dynamical and mechanistic. We assume the transformed signal, 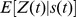, to be an invertible function of 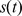. The aggregate behavior of the two types of error determines the signaling fidelity, and ignoring either may cause erroneous conclusions. We interpret 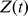 as the current cellular estimate or ‘readout’ of the faithfully transformed signal. The magnitude of the fidelity error relative to the variance in 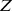, Eq. 7, is a dimensionless measure of the quality of that estimate since 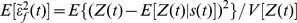. Furthermore, we have shown that 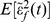 is related to the mutual information between the input and output [7].

To apply our approach experimentally, we can use microfluidic technology to expose cells to the same controlled but time-varying input in the medium [33], and a fluorescent reporter to monitor the network output, 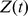. This reporter could measure, for example, a level of gene expression or the extent of translocation of a transcription factor. The transformed signal, 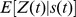, and its variance (for a given probability distribution of the input process) can then be estimated with sufficient amounts of data by monitoring 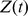 in each cell and 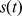 in the microfluidic medium. We can determine the mechanistic error by measuring the average squared difference between the output of one cell and that of another — because the outputs of two cells are conjugate given the history of the input [7] –and hence determine the dynamical error by applying Eq. 6.

Our analysis is complementary to one based on information theory and the entire distribution of input and output [7]. Without making strong assumptions about the network and the input, calculation of mutual information is challenging for dynamic inputs. Previous work has considered either the mutual information between entire input and output trajectories with a Gaussian joint distribution of input and output [19], [34], or the ‘instantaneous’ mutual information between input and output at time 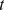 [19] (applicable in principle to non-Gaussian settings). Our approach, however, depends only on conditional moments and avoids the need to fully specify the distribution of the input process, which is often poorly characterized.

The environments in which cells live are inherently dynamic and noisy. Here we have developed mathematical techniques to quantify how cells interpret and respond to fluctuating signals given their stochastic biochemistry. Our approach is general and will help underpin studies of cellular behavior in natural, dynamic environments.

## Methods

### Orthogonality of transformed signal, dynamical error and mechanistic error

Define 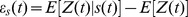, the transformed signal with zero mean. Then the signal and error components of Eq. 5 are pairwise uncorrelated:
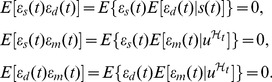
(14)


### Orthogonal decomposition of a random variable based on a filtration

Eq. 5 is a special case of the following general decomposition for any random variable (with finite expectation), here denoted 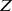. Consider a filtration, or increasing sequence of conditioning ‘information sets’, 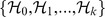, where 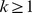 and 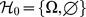. Let 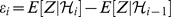 for 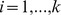, and let 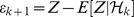. Then the decomposition
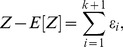
(15)satisfies 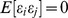 for all 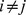 since the sequence 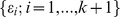 is a martingale difference sequence with respect to the filtration (SI). Therefore, 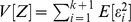.

## Supporting Information

Text S1 The complete supporting information is provided as Text S1.
(PDF)
