# Supplementary material for: The Fidelity of Dynamic Signaling by Noisy Biomolecular Networks
Source: PLoS Comput Biol. 2013 Mar 28;9(3):e1002965. doi: 10.1371/journal.pcbi.1002965 (PMC3610653; doi:10.1371/journal.pcbi.1002965)
Supplement: Text S1 — The complete supporting information is provided as Text S1. (PDF) [file pcbi.1002965.s001.pdf]

The fidelity of dynamic signaling by noisy biomolecular networks: Supplementary Information

Clive G. Bowsher,* Margaritis Voliotis & Peter S. Swain†

A brief primer on conditional expectations

Conditional expectations are not commonly used outside of probability and statistics. We present here a short introduction and list of their properties.

For a random variable Z and a random vector X, the conditional expectation $E[Z|X]$ is itself a random variable because it is a function of the random variables in X. For continuous random variables, $E[Z|X]$ is defined as

$$E[Z|X = x] = \int dz \, z \, p(z|x) \tag{16}$$

where $p(z|x)$ is the conditional probability density of Z given X. It satisfies $p(z|x) = p(z, x)/p(x)$, by Bayes's rule.

Let Z be any random variable and X any random vector. Among all random variables that are a function of X, the conditional expectation is the unique random variable which minimizes the mean squared error of approximating Z using X alone. That is,

$$E\Big[\Big(Z - f(X)\Big)^2\Big] \geq E\Big[\Big(Z - E[Z|X]\Big)^2\Big], \tag{17}$$

with equality if and only if $f(X) = E[Z|X]$ (with probability one). We can interpret $E[(Z - f(X))^2]$ as the magnitude of the fidelity error for the approximation (or representation) of Z by $f(X)$.

The above property applies not just to conditioning on a random vector, but to any conditioning information. For example, let $u^{\mathcal{H}_t}$ be the (internal) history of a continuous time stochastic process, u. Then, among all random variables that are (measurable) functions of the trajectory of u up to time t, the conditional expectation $E[Z|u^{\mathcal{H}_t}]$ minimises the mean squared error of approximating Z using only the history of u. The minimum mean squared error (MMSE) is therefore given by $E[(Z - E[Z|u^{\mathcal{H}_t}])^2] = E[V[Z|u^{\mathcal{H}_t}]]$.

The conditional expectation has the following properties for any two random variables Z, Y and any random vector X:

(i) If Z and X are independent, then

$$E[Z|X] = E[Z].$$

*Joint corresponding author, c.bowsher@bristol.ac.uk
†Joint corresponding author, peter.swain@ed.ac.uk

(ii) If Z and Y are conditionally independent given X, then

$$E[Z|Y,X] = E[Z|X].$$

(iii) For real constants a and b

$$E[aZ + bY|X] = aE[Z|X] + bE[Y|X].$$

(iv) If knowing the random vector X implies that Y is known then

$$E[ZY|X] = YE[Z|X],$$

and so $E[g(X)|X] = g(X)$ for any (measurable) function $g(X)$.

(v) If knowing X implies that Y is known then

$$E[Z|Y] = E\Big[E[Z|X]\Big|Y\Big].$$

(vi) For any X,

$$E\Big[E[Z|X]\Big] = E[Z].$$

General orthogonal decomposition into signal and fidelity error components

We are interested in how the dynamics of a network output Z are influenced by several stochastic 'variables', denoted $\mathcal{X}_1, \mathcal{X}_2, ..., \mathcal{X}_k$ ($k \geq 1$). Each $\mathcal{X}_i$ will play the role of conditioning information and, formally, is the σ-field generated by a random variable, random vector or a continuous-time stochastic process. For a network input, $\mathcal{X}_i$ may pertain to a non-dynamic input, a finite-dimensional summary (or 'statistic') of a dynamic input process up to time t, or the history at time t of a continuous-time process. For biochemical species intrinsic (or internal) to the signal transduction network and for purely confounding extrinsic processes (e.g. the number of ribosomes), $\mathcal{X}_i$ is typically the history of the number of molecules of the species up to time t. For example, we might want to decompose mechanistic error into components due to different 'modules' in a signaling cascade and due to confounding extrinsic fluctuations [1].

We provide a proof of the orthogonality of the following general decomposition of the random variable $Z(t)$:

$$Z(t) = E[Z(t)|\mathcal{X}_1] + \sum_{i=2}^{k} \varepsilon_i(t) + \{Z(t) - E[Z(t)|\mathcal{X}_1, \mathcal{X}_2, ..., \mathcal{X}_k]\},\ t \geq 0, \tag{18}$$

where $\varepsilon_i(t) := E[Z(t)|\mathcal{X}_1, \mathcal{X}_2, ..., \mathcal{X}_i] - E[Z(t)|\mathcal{X}_1, \mathcal{X}_2, ..., \mathcal{X}_{i-1}]$ for $i = 2, ..., k$, and we define $\varepsilon_{k+1}(t) := Z(t) - E[Z(t)|\mathcal{X}_1, \mathcal{X}_2, ..., \mathcal{X}_k]$, and $\varepsilon_1(t) := E[Z(t)|\mathcal{X}_1]$.

Specifically, we show that the covariance is zero for every pair of random variables (r.v.'s) on the right-hand side of Eq. 18. It then follows that the the variance of $Z(t)$ is given by the variance of the sum of these r.v.'s. For those familiar with martingale difference sequences, we then provide a more concise

proof of the orthogonality. Notice that $E[\varepsilon_i(t)] = 0$ for $i = 2, ..., k+1$. We need to consider the covariance of all possible pairs. We do this in 4 stages.

First, we show that $\text{Cov}[\varepsilon_i(t), \varepsilon_j(t)] = E[\varepsilon_i(t)\varepsilon_j(t)] = 0$ for $2 \le i < j \le k$.

$$\begin{aligned} E[\varepsilon_i(t)\varepsilon_j(t)] &= E\{E[\varepsilon_i(t)\varepsilon_j(t)|\mathcal{X}_1, \mathcal{X}_2, ..., \mathcal{X}_i]\} \\ &= E\{\varepsilon_i(t)E[(E[Z(t)|\mathcal{X}_1, \mathcal{X}_2, ..., \mathcal{X}_j] - E[Z(t)|\mathcal{X}_1, \mathcal{X}_2, ..., \mathcal{X}_{j-1}])|\mathcal{X}_1, \mathcal{X}_2, ..., \mathcal{X}_i]\} \\ &= E\{\varepsilon_i(t)[E[Z(t)|\mathcal{X}_1, \mathcal{X}_2, ..., \mathcal{X}_i] - E[Z(t)|\mathcal{X}_1, \mathcal{X}_2, ..., \mathcal{X}_i]]\} = 0, \end{aligned} \tag{19}$$

where the last line follows because $i \le j-1$ and hence $(\mathcal{X}_1, \mathcal{X}_2, ..., \mathcal{X}_i) \subseteq (\mathcal{X}_1, \mathcal{X}_2, ..., \mathcal{X}_{j-1})$.

Second, we show that $\text{Cov}[\varepsilon_i(t), \varepsilon_{k+1}(t)] = E[\varepsilon_i(t)\varepsilon_{k+1}(t)] = 0$ for $2 \le i \le k$. Reasoning similarly to the first case,

$$\begin{aligned} E[\varepsilon_i(t)\varepsilon_{k+1}(t)] &= E\{\varepsilon_i(t)E[(Z(t) - E[Z(t)|\mathcal{X}_1, \mathcal{X}_2, ..., \mathcal{X}_{k+1}])|\mathcal{X}_1, \mathcal{X}_2, ..., \mathcal{X}_i]\} \\ &= E\{\varepsilon_i(t)[E[Z(t)|\mathcal{X}_1, \mathcal{X}_2, ..., \mathcal{X}_i] - E[Z(t)|\mathcal{X}_1, \mathcal{X}_2, ..., \mathcal{X}_i]]\} = 0. \end{aligned} \tag{20}$$

Third, we show that $\text{Cov}[\varepsilon_1(t), \varepsilon_j(t)] = E\{(\varepsilon_1(t) - E[Z(t)])\varepsilon_j(t)\} = 0$ for $2 \le j \le k$. Again, with a similar line of reasoning but conditioning on $\mathcal{X}_1$,

$$E\{(\varepsilon_1(t) - E[Z(t)])\varepsilon_j(t)\} = E\{(\varepsilon_1(t) - E[Z(t)])E[\varepsilon_j(t)|\mathcal{X}_1]\} = 0.$$

Fourth, we show that $\text{Cov}[\varepsilon_1(t), \varepsilon_{k+1}(t)] = E\{(\varepsilon_1(t) - E[Z(t)])\varepsilon_{k+1}(t)\} = 0$.

$$E\{(\varepsilon_1(t) - E[Z(t)])\varepsilon_{k+1}(t)\} = E\{(\varepsilon_1(t) - E[Z(t)])E[\varepsilon_{k+1}(t)|\mathcal{X}_1]\} = 0.$$

More concisely, we can write

$$Z(t) - E[Z(t)] = \{\varepsilon_1(t) - E[Z(t)|\mathcal{X}_0]\} + \sum_{i=2}^{k+1} \varepsilon_i(t), \tag{21}$$

where $\mathcal{X}_0 = \{\Omega, \varnothing\}$ and therefore $\tilde{\varepsilon}_1(t) := \{\varepsilon_1(t) - E[Z(t)|\mathcal{X}_0]\} = \varepsilon_1(t) - E[Z(t)]$. To establish the orthogonality of the decomposition of $Z(t)$, it suffices to notice that the sequence of r.v.'s $\{\tilde{\varepsilon}_1(t), \varepsilon_2(t), ..., \varepsilon_{k+1}(t)\}$ is a Martingale Difference Sequence (MDS) with respect to the filtration $\{\mathcal{X}_0, (\mathcal{X}_0, \mathcal{X}_1), (\mathcal{X}_0, \mathcal{X}_1, \mathcal{X}_2), ..., (\mathcal{X}_0, ..., \mathcal{X}_k)\}$. This is because the ith r.v. in the sequence has conditional expectation equal to zero given the ith term in the filtration, for all $i = 1, ..., (k+1)$. It follows immediately from the fact that the sequence is a MDS that each pair of its constituent r.v.'s is uncorrelated and therefore has zero covariance.

Finally, notice that $E[\varepsilon_{k+1}(t)^2] = E\{V[Z(t)|\mathcal{X}_1, \mathcal{X}_2, ..., \mathcal{X}_k]\}$ and that for $i = 2, ..., k$,

$$\begin{aligned} E[\varepsilon_i(t)^2] &= E\{E[(E[Z(t)|\mathcal{X}_1, \mathcal{X}_2, ..., \mathcal{X}_i] - E[Z(t)|\mathcal{X}_1, \mathcal{X}_2, ..., \mathcal{X}_{i-1}])^2|\mathcal{X}_1, \mathcal{X}_2, ..., \mathcal{X}_{i-1}]\} \\ &= E\{V[E[Z(t)|\mathcal{X}_1, \mathcal{X}_2, ..., \mathcal{X}_i]|\mathcal{X}_1, \mathcal{X}_2, ..., \mathcal{X}_{i-1}]\}. \end{aligned} \tag{22}$$

Taken together, the above results also complement the general variance decomposition for dynamic systems in [1]. They provide an underlying, orthogonal decomposition of the process $Z(t)$ itself. The

component processes have variances equal to the components of the variance decomposition in [1].

An exact, analytical approach to the dynamics of stochastic biomolecular networks with fluctuating inputs

We show here how to analyse gene expression regulated by fluctuating inputs. The essence of our approach is to analyse the dynamics of the system in the presence of 'fixed' trajectories of the time-varying propensities. This provides expressions for the conditional moments of the output $Z(t)$ as a function of the realised history of the stochastic inputs, revealing how the expected network output responds in continuous time to the fluctuating inputs.

Birth-death process with fluctuating inputs

We begin with the simple but biologically relevant example in which a single molecular species is synthesised and degraded in response to fluctuating inputs. We denote by $M(t)$ the number of molecules of that species at time t. The master equation with time-varying propensity for molecular births, $u(t)$, and for deaths, $d(t)$, is given by

$$\frac{dP_i(t)}{dt} = u(t)P_{i-1}(t) + d(t)(i+1)P_{i+1}(t) - [u(t) + id(t)]P_i(t), \tag{23}$$

for $i = 1, 2, \ldots$, with $P_i(t)$ the probability that $M(t)$ equals i. When the propensities are deterministic functions of time, Eq. 23 can be solved to obtain a bivariate system of differential equations for the first and second moments. This does not appear to deal with stochastic inputs. Suppose however that the inputs are exogenous processes: their future paths may depend on their own history but are (conditionally) independent of the history of M. (More precisely, $u^{\mathcal{H}_\infty} \perp\!\!\!\perp M^{\mathcal{H}_t} | u^{\mathcal{H}_t}$). The signal u then acts as a 'pure input' to the system. Under this condition, the same system of differential equations describes the first and second *conditional* moments, where the conditioning is on the history of the inputs at time t denoted $(u, d)^{\mathcal{H}_t}$, together with the initial condition M_0 (subscripts indexing time):

$$\frac{dE[M_t|(u,d)^{\mathcal{H}_t}, M_0]}{dt} = u_t - d_t E[M_t|(u,d)^{\mathcal{H}_t}, M_0], \tag{24}$$

and

$$\begin{aligned} \frac{dE[M_t^2|(u,d)^{\mathcal{H}_t}, M_0]}{dt} &= u_t + E[M_t|(u,d)^{\mathcal{H}_t}, M_0] \\ &\cdot [2u_t + d_t] - 2d_t E[M_t^2|(u,d)^{\mathcal{H}_t}, M_0]. \end{aligned} \tag{25}$$

The conditioning on the initial number of mRNAs should not be omitted if $M(0)$ is non-deterministic, for example when analysing the (exactly) stationary system. A single birth-death process with fluctuating inputs and the associated conditional master equation is considered in the Supporting Information of [2] but the authors do not solve for the individual first and second conditional moments or for the extrinsic variance component.

Conditional and unconditional dynamics of gene expression with a fluctuating transcriptional input

We will write $M(t)$ and $Z(t)$ for the number of molecules of mRNA and of protein respectively. Eq. 23 describes the conditional dynamics of mRNA (with $d(t)$ set to its constant degradation rate, d_M). Due to the hierarchichal structure of the model, protein numbers $Z(t)$ also follow a birth-death process with the exogenous input $M(t)v$ (where v is the rate constant for the translation reaction).

We will analyse the (second-order) stationary gene expression system with fluctuating, exogenous transcriptional input $u(t)$. We do not assume a deterministic initial condition. An assumption is needed on the 'memory' of the fluctuations in u. We assume that the expected past (and future) deviation of u from its mean decreases exponentially in time and is related to the current deviation at time t by:

$$E[u_r - E\{u\}|u_t] = (u_t - E\{u\})e^{-d_u|t-r|}, \tag{26}$$

hence $\mathrm{Corr}(u_t, u_r) = \exp(-d_u|t-r|)$. Strictly, we only use that Eq. 26 holds for $r < t$. Eq. 26 is satisfied, for example, for all r, t for stationary random telegraph (2-state Markov chain) and birth-death processes.

Solving Eq. 24 for the dynamics of the first conditional moment of mRNA levels yields

$$E[M_t|u^{\mathcal{H}_t}, M_0] = M_0 e^{-d_M t} + \int_0^t u(s)e^{-d_M(t-s)}ds. \tag{27}$$

We can take the (unconditional) expectation of both sides of this equation (interchanging the order of the expectation and integration with respect to time) to find that the stationary mean of mRNA is given by $E[M_t] = E\{E[M_t|u^{\mathcal{H}_t}, M_0]\} = E[u_t]/d_M$.

Notice that, for all $t > 0$, $E[Z_t|u^{\mathcal{H}_t}, M^{\mathcal{H}_t}, M_0, Z_0] = E[Z_t|u^{\mathcal{H}_t}, M^{\mathcal{H}_t}, M_0, Z_0]$ because $Z^{\mathcal{H}_t} \perp\!\!\!\perp u^{\mathcal{H}_t} | M^{\mathcal{H}_t}, M_0, Z_0$. The differential equations analogous to Eq. 24 and 25 for the dynamics of the first and second conditional moments of protein levels are thus

$$\frac{dE[Z_t|u^{\mathcal{H}_t}, M^{\mathcal{H}_t}, M_0, Z_0]}{dt} = vM_t - d_Z E[Z_t|u^{\mathcal{H}_t}, M^{\mathcal{H}_t}, M_0, Z_0],$$

and

$$\frac{dE[Z_t^2|u^{\mathcal{H}_t}, M^{\mathcal{H}_t}, M_0, Z_0]}{dt} = vM_t + E[Z_t|u^{\mathcal{H}_t}, M^{\mathcal{H}_t}, M_0, Z_0][2vM_t + d_Z] - 2d_Z E[Z_t^2|u^{\mathcal{H}_t}, M^{\mathcal{H}_t}, M_0, Z_0].$$

Solving these yields

$$E[Z_t|u^{\mathcal{H}_t}, M^{\mathcal{H}_t}, M_0, Z_0] = Z_0 e^{-d_Z t} + \int_0^t vM_s e^{-d_Z(t-s)}ds, \tag{28}$$

and

$$E[Z_t^2|u^{\mathcal{H}_t}, M^{\mathcal{H}_t}, M_0, Z_0] = Z_0^2 e^{-2d_Z t} + \int_0^t \{vM_s + E[Z_s|u^{\mathcal{H}_s}, M^{\mathcal{H}_s}, M_0, Z_0][2vM_s + d_Z]\}e^{-2d_Z(t-s)}ds. \tag{29}$$

To derive Eq. 9 (main text) we use the relationship $E[Z(t)|u^{\mathcal{H}_t}, M_0, Z_0] = E[E[Z(t)|M^{\mathcal{H}_t}, Z_0]|u^{\mathcal{H}_t}, M_0, Z_0]$

and Eq. 28. We thus find that

$$\begin{aligned} E[Z(t)|u^{\mathcal{H}_t}, M_0, Z_0] &= Z_0 e^{-d_Z t} + \int_0^t vE[M_s|u^{\mathcal{H}_s}, M_0]e^{-d_Z(t-s)}ds \\ &= Z_0 e^{-d_Z t} + \frac{vM_0}{(d_Z - d_M)}(e^{-d_M t} - e^{-d_Z t}) + v\int_0^t \int_0^s u_r e^{-d_Z t + (d_Z - d_M)s + d_M r} dr ds \\ &\triangleq f_1(Z_0, t) + f_2(M_0, t) + v\int_0^t f_3(s; u(r), r \le s)ds, \end{aligned} \tag{30}$$

where the second line follows from Eq. 27 and the exogeneity of the input (and the third line defines the functions f_1, f_2 and the functional f_3). Eq. 9 is the special case $M_0 = Z_0 = 0$.

We take the expectation of Eq. 28 to find that the stationary mean protein level is given by $E[Z_t] = vE[M_t]/d_Z$. To derive the stationary variance of protein levels we use Eq. 29 and the relationship $E[Z_t^2] = E\{E[Z_t^2|u^{\mathcal{H}_t}, M^{\mathcal{H}_t}, M_0, Z_0]\}$. We thus find, since $M_s E[Z_s|u^{\mathcal{H}_s}, M^{\mathcal{H}_s}, M_0, Z_0] = E[M_s Z_s|u^{\mathcal{H}_s}, M^{\mathcal{H}_s}, M_0, Z_0]$ and the unconditional moments involved are time-invariant in this stationary setting, that

$$E[Z_t^2] = E[Z_t] + vE[M_t Z_t]/d_Z. \tag{31}$$

Now $E[M_t Z_t] = E\{M_t E[Z_t|M^{\mathcal{H}_t}, M_0, Z_0]\}$ and hence by Eq. 28,

$$E[M_t Z_t] = e^{-d_Z t}E[M_t Z_0] + v\int_0^t E[M_t M_s]e^{-d_Z(t-s)}ds. \tag{32}$$

Furthermore, $E[M_t M_s] = E\{M_s E[M_t|M_s, u^{\mathcal{H}_t}]\}$, where $E[M_t|M_s, u^{\mathcal{H}_t}]$ is given by the analogue of Eq. 27 in which s rather than zero is the initial time. To fund the autocovariance of mRNA, we will thus need the moment $E[M_s u_r]$ for $r > s \ge 0$. Notice that $E[M_s u_r] = E\{u_r E[M_s|u^{\mathcal{H}_s}, M_0]\}$ with the conditional moment given by Eq. 27, since the exogeneity of the input implies that $M_s \perp\!\!\!\perp u^{\mathcal{H}_r}|u^{\mathcal{H}_s}, M_0$. We find that in the stationary case,

$$E[M_s u_r] = E[M]E[u] + \text{Cov}(u_r, u_s)/(d_u + d_M), \tag{33}$$

(where we have used that $E[M_s u_r]$ must depend only on $(r - s)$ and therefore taken the limit of $E[M_s u_r]$ as $r, s \to \infty$ holding $(r - s)$ constant). It follows that

$$E[M_t M_s] = E[M_t^2]e^{-d_M|t-s|} + \frac{E[u]^2}{d_M^2}(1 - e^{-d_M|t-s|}) + \frac{V[u]}{(d_M - d_u)(d_M + d_u)}(e^{-d_u|t-s|} - e^{-d_M|t-s|}).$$

Combining this expression for $E[M_t M_s]$ with Eq. 32 (after taking the limit of $E[M_t Z_t]$ as $t \to \infty$, which is justified since $E[M_t Z_t]$ is time-invariant), and substituting into Eq. 31 yields $V[Z_t]$, using that $V[Z_t] = E[Z_t^2] - E[Z_t]^2$. We find $V[Z_t]$ as given by the sum of the 3 variance components in Eqs. 11, 12, and 76 (main text).

Quantifying fidelity errors by evaluating the variance components analytically

We first provide an outline of the main steps used to evaluate the magnitudes of the fidelity errors, given that we have now derived $V[Z_t]$ (see previous section of SI).

1. Derive $V\{E[Z(t)|u(t)]\}$ using $E[Z(t)|u(t)] = E[E[Z(t)|u^{\mathcal{H}_t}, M(0), Z(0)]|u(t)]$. We find, as expected from Eq. 10, that $V\{E[Z(t)|u(t)]\} = V[u]v^2/(d_u + d_M)^2(d_u + d_Z)^2$. The magnitude of the total fidelity error $\varepsilon_f(t) = \varepsilon_d(t) + \varepsilon_m(t)$ can now be quantified as equal to the difference $V[Z(t)] - V\{E[Z(t)|u(t)]\}$.

2. Derive $E[\varepsilon_m(t)^2]$ using Eq. 9 and step 1. above. Without altering the essence of the interpretations of the error components, we can make the analysis considerably more tractable by conditioning also on the initial condition, for example defining $\varepsilon_m(t) = Z(t) - E[Z(t)|u^{\mathcal{H}_t}, M(0), Z(0)]$. The steady-state magnitude of the mechanistic error is found as $\lim_{t\to\infty} E\{V[Z(t)|u^{\mathcal{H}_t}, M(0), Z(0)]\} = E[Z^2] - \lim_{t\to\infty} E\{E[Z(t)|u^{\mathcal{H}_t}, M(0), Z(0)]^2\}$, where $E[Z(t)|u^{\mathcal{H}_t}, M(0), Z(0)]$ is derived as for Eq. 9 (but without assuming $M(0) = Z(0) = 0$).

3. Find $E[\varepsilon_d(t)^2] = V[Z(t)] - V\{E[Z(t)|u(t)]\} - E[\varepsilon_m(t)^2]$ using the results of steps 1. to 3. above.

Step 1. From Eq. 30 and the relationship $E[Z_t|u_t] = E[E[Z_t|u^{\mathcal{H}_t}]|u_t]$ we obtain

$$\begin{aligned} E[Z_t|u_t] &= \left\{E[E[Z_0|u_0]|u_t]e^{-d_Z t} + \frac{vE[E[M_0|u_0]|u_t]}{(d_Z - d_M)}(e^{-d_M t} - e^{-d_Z t})\right\} + \\ &\quad \left\{v\int_0^t\int_0^s E[u_r|u_t]e^{-d_Z t + (d_Z - d_M)s + d_M r}drds\right\} \\ &\triangleq m_1(u_t, t) + m_2(u_t, t). \end{aligned} \tag{34}$$

with $E[u_r|u_t]$ given by Eq. 26. The expression for $E[Z_t|u_t]$ in Eq. 10 (main text) then follows by setting $M_0 = Z_0 = 0$ and letting $t \to \infty$. We want to derive $V\{E[Z(t)|u(t)]\}$ using Eq. 34. Since $u(t)$ is stationary, the second moment $V\{E[Z(t)|u(t)]\}$ is time-invariant. A valid approach is therefore to take the variance of Eq. 34 and then take the limit of the resultant expression as $t \to \infty$. The subsequent derivation is quite subtle.

Notice that $V\{E[Z(t)|u(t)]\}$ depends only on the variances and covariance of $m_1(u_t, t)$ and $m_2(u_t, t)$, moments which depend on u_t via its *time-invariant* (stationary) distribution. Let U be some r.v. with that same distribution. We then see that $\lim_{t\to\infty} V[m_1(U, t)] = 0$. Furthermore, provided that $m_2(U, t)$ is bounded by an integrable r.v. for all t (physically a weak assuption), then $\lim_{t\to\infty} V[m_2(U, t)] = V[\lim_{t\to\infty} m_2(U, t)]$. Similarly, provided that $m_1(U, t)m_2(U, t)$ is bounded by an integrable r.v. for all t (physically a weak assuption), then $\lim_{t\to\infty} \mathrm{Cov}[m_1(U, t), m_2(U, t)] = 0$. Putting all this together yields $V\{E[Z(t)|u(t)]\} = V[\lim_{t\to\infty} m_2(U, t)] = V[u]v^2/(d_u + d_M)^2(d_u + d_Z)^2$, as stated in Eq. 11 (main text).

Step 2. As explained in the main text, the steady-state magnitude of the intrinsic noise is equal to $\lim_{t\to\infty} E\{V[Z_t|u^{\mathcal{H}_t}, M_0, Z_0]\} = E[Z^2] - \lim_{t\to\infty} E\{E[Z_t|u^{\mathcal{H}_t}, M_0, Z_0]^2\}$, where $E[Z_t|u^{\mathcal{H}_t}, M_0, Z_0]$ is given by Eq. 30 and we derived $E[Z^2]$ in the previous section to find the total variance of protein levels. It follows from Eq. 30, using the notation established there, that

$$E\{E[Z_t|u^{\mathcal{H}_t}, M_0, Z_0]^2\} = E\left\{\left[f_1(Z_0, t) + f_2(M_0, t) + v\int_0^t f_3(s; u(r), r \le s)ds\right]^2\right\}.$$

The right-hand side is clearly the sum of 6 expected values. On taking the limit as $t \to \infty$, all but one of these 6 terms converges to zero. We do not go into lengthy details concerning the other 5 terms, but note

that their evaluation requires the (stationary) moments $E[Z_t^2]$, $E[M_t^2]$, $E[M_tZ_t]$, $E[M_0u_t]$ and $E[Z_0u_t]$. The last of these has not been derived above. Notice that $E[Z_0u_t] = E\{Z_0E[u_t|u_0, Z_0]\} = E\{Z_0E[u_t|u_0]\}$, where we have used the exogeneity of the input u and $E[u_t|u_0]$ is given by 26.

The only non-zero term in the limit is given by

$$\begin{aligned}\lim_{t\to\infty} v^2 E\left\{\left[\int_0^t f_3(s)ds\right]^2\right\} &= \lim_{t\to\infty} v^2 E\left\{\int_0^t\int_0^t f_3(s_1)f_3(s_2)ds_1ds_2\right\} \\ &= \lim_{t\to\infty} v^2 \int_0^t\int_0^t\int_0^{s_2}\int_0^{s_1} E\{u_{r_1}u_{r_2}\}e^{-2d_Zt+(d_Z-d_M)(s_1+s_2)+d_M(r_1+r_2)}du_1du_2ds_1ds_2 \\ &= E[Z_t^2] + \frac{V[u]v^2(d_u+d_M+d_Z)}{d_Zd_M(d_{Z+}d_M)(d_u+d_M)(d_u+d_Z)},\end{aligned} \tag{35}$$

where we have used that the autocorrelation of u implied by Eq. 26 gives $E\{u_{r_1}u_{r_2}\} = E[u]^2 + V[u]e^{-d_u|r_2-r_1|}$. Evaluation of the multiple integral in Eq. 35 requires some care, being careful to distinguish when $s_1 \leq s_2$ and $s_2 \leq s_1$.

Step 3. is immediate

Modeling promoter activity

To model the propensity of the transcription reaction, we follow the approach based on statistical mechanics and pioneered by Ackers *et al.* [3] to calculate the transcriptional propensity, r. We assume that an input $\hat{u}(t)$ acts immediately as a transcriptional activator. For example, $\hat{u}(t)$ could be the level of a hormone that directly binds and activates a transcription factor that is itself pre-bound to the promoter of the target gene. The input $u(t)$, which we have defined as a rate, is then proportional to $w\hat{u}(t)$, where w is the rate of transcription when the promoter is in an active, transcription-ready state. In the notation that follows, we do not explicitly write the time dependence of any random variables.

No feedback

We assume that the promoter can be bound by RNA polymerase, denoted P, and by the input $\hat{u}$:

$$r_{\text{no}} = w\frac{K_PP + K_{P,u}P\hat{u}}{1 + K_PP + K_u\hat{u} + K_{P,u}P\hat{u}}, \tag{36}$$

where the K_i are DNA binding affinities (association constants). The transcriptional propensity is proportional to the probability at time t that the DNA is bound by RNA polymerase. If we further assume that transcription occurs only in the presence of $\hat{u}$, then

$$r_{\text{no}} = w\frac{K_{P,u}P\hat{u}}{1 + K_PP + K_u\hat{u} + K_{P,u}P\hat{u}}, \tag{37}$$

which simplies to

$$r_{\text{no}} \simeq w\frac{K_{P,u}P}{1 + K_PP}\hat{u}, \tag{38}$$

if $\hat{u}$ binds weakly so that $1 + K_P P \gg (K_u + K_{P,u} P)\hat{u}$. For a constant level of polymerase P, Eq. 38 is the form we use in the two-stage model of gene expression without feedback by absorbing the constant prefactor into the definition for u: $u(t) = w\frac{K_{P,u}P}{1+K_P P}\hat{u}(t)$.

Negative autoregulatory feedback

If Z, the protein coded by the gene, is a repressor and can bind to its own promoter then

$$r_{\mathrm{neg}} = w\frac{K_P P + K_{P,u} P\hat{u}}{1 + K_P P + K_u\hat{u} + K_{P,u}P\hat{u} + K_Z Z}. \tag{39}$$

Assuming that transcription occurs only in the presence of $\hat{u}$ and that $1 + K_p P \gg (K_u + K_{P,u})\hat{u}$ as before, then

$$\begin{aligned} r_{\mathrm{neg}} &\simeq w\frac{K_{p,u}P\hat{u}}{1 + K_P P + K_Z Z} \\ &= w\frac{\frac{K_{P,u}P}{1+K_P P}\hat{u}}{1 + \frac{K_Z}{1+K_P P}Z}. \end{aligned} \tag{40}$$

Eq. 40 is used in the main text with $K_1 = \frac{K_Z}{1+K_P P}$ and $u(t) = w\frac{K_{P,u}P}{1+K_P P}\hat{u}(t)$. We note that this definition of $u(t)$ is consistent with the no feedback case and that $u(t)$ is a rate (with units given by the reciprocal of time).

Positive autoregulatory feedback

If Z is an activator and binds to its own promoter then

$$r_{\mathrm{pos}} = w\frac{K_P P + K_{P,u}P\hat{u} + K_{P,Z}PZ + K_{P,u,Z}P\hat{u}Z}{1 + K_P P + K_u\hat{u} + K_{P,u}P\hat{u} + K_Z Z + K_{P,Z}PZ + K_{u,Z}\hat{u}Z + K_{P,u,Z}P\hat{u}Z}. \tag{41}$$

If $\hat{u}$ is required for transcription and $1 + K_P P \gg (K_u + K_{P,u})\hat{u}$ then

$$\begin{aligned} r_{\mathrm{pos}} &\simeq w\frac{K_{P,u}P\hat{u} + K_{P,Z}PZ + K_{P,u,Z}P\hat{u}Z}{1 + K_P P + K_Z Z + K_{P,Z}PZ + K_{u,Z}\hat{u}Z + K_{P,u,Z}P\hat{u}Z} \\ &\simeq w\frac{K_{P,u}P\hat{u} + K_{P,Z}PZ + K_{P,u,Z}P\hat{u}Z}{1 + K_P P + K_Z Z + K_{P,Z}PZ} \end{aligned} \tag{42}$$

if further $1 + K_P P \gg (K_{u,Z} + K_{P,u,Z}P)\hat{u}Z$ also holds. Finally, we assume that Z and $\hat{u}$ cannot simultaneously bind to the promoter

$$\begin{aligned} r_{\mathrm{pos}} &\simeq w\frac{K_{P,u}P\hat{u} + K_{P,Z}PZ}{1 + K_P P + K_Z Z + K_{P,Z}PZ} \\ &= w\frac{\frac{K_{P,u}P}{1+K_P P}\hat{u} + \frac{K_{P,Z}P}{1+K_P P}Z}{1 + \frac{K_Z + K_{P,Z}P}{1+K_P P}Z} \\ &= \frac{u + wK_1 Z}{1 + (K_1 + K_2)Z} \end{aligned} \tag{43}$$

with $K_1 = \frac{K_{P,Z}P}{1+K_P P}$, $K_2 = \frac{K_Z}{1+K_P P}$, and $u(t) = w\frac{K_{P,u}P}{1+K_P P}\hat{u}(t)$.

Quantifying fidelity errors using Langevin theory

Using Langevin theory [4], we can derive approximate expressions for the fidelty errors and their magnitudes. Our approximation improves the closer the system is to both stationary-state and the 'deterministic' high copy number limit.

Modeling the fluctuating input

We model the input $u(t)$ as an Ornstein-Uhlenbeck (OU) process. A Gaussian OU process is specified entirely by its mean, variance, and autocorrelation time. It is only these properties of the input that determine the magnitudes of the fidelity errors in our exact solution for the two-stage model of gene expression.

We use a stochastic differential equation to describe the dynamics of u:

$$\dot{u} = -(u_t - E[u])d_U + \xi_1(t), \tag{44}$$

where $\xi_1(t)$ is a white noise term that satisfies

$$E[\xi_1(t)\xi_1(t')] = 2d_U V[u]\delta(t-t'), \tag{45}$$

using the Kronecker delta, and has $E[\xi_1] = 0$.

Modeling the biochemical network

Within Langevin theory, the dynamics of a biochemical system are modelled as a set of coupled stochastic differential equations [5]. If we let $\mathbf{X}$ be a vector, with X_i the copy number of the ith chemical species in the network, and assume that the dynamics of the network tend to a stationary state, then we can linearize the differential equations around that stationary state to give

$$\dot{\mathbf{X}} = \mathbf{A}\left(\mathbf{X_t} - E[\mathbf{X}]\right) + \xi(t), \tag{46}$$

where $\mathbf{A}$ is the Jacobian of the system evaluated at the stationary mean concentrations, $E[\mathbf{X}]$. The ξ_i are stochastic variables with $E[\xi_i] = 0$ and

$$E[\xi_i(t)\xi_j(t')] = \Gamma_{ij}\delta(t-t'), \tag{47}$$

where the diffusion matrix Γ_{ij} describes the fluctuations in the system and is related to the stoichiometric matrix, $\mathbf{S}$, and the propensities of each reaction, $\mathbf{p}$, by $\mathbf{\Gamma} = \mathbf{S}\,\mathrm{diag}(\mathbf{p})\,\mathbf{S}^T$ [6].

This approximate system can be solved using linear algebra [7]. Let B_{ij} be an eigenvector of $\mathbf{A}$ with eigenvalue $\lambda^{(j)}$ such that

$$\sum_j A_{ij}B_{jk} = \lambda^{(k)}B_{ik}. \tag{48}$$

Then we have

$$X_i(t) = E[X_i] + \sum_{j,k} B_{ij} \mathrm{e}^{\lambda^{(j)} t} B^{-1}_{jk} (X_k(0) - E[X_k]) + \sum_{j,k} \int_0^t ds \, B_{ij} \mathrm{e}^{\lambda^{(j)}(t-s)} B^{-1}_{jk} \xi_k(s), \quad (49)$$

for the dynamics of $X_i(t)$ near steady-state. The autocorrelation function is

$$\begin{aligned} &E[X_i(t_1)X_j(t_2)] - E[X_i]E[X_j] \\ &\quad = \sum_{p,q,r,s} B_{ip} B_{jr} \frac{\Gamma_{qs}}{\lambda^{(p)} + \lambda^{(r)}} \left[\mathrm{e}^{\lambda^{(p)} t_1 + \lambda^{(r)} t_2} - \mathrm{e}^{\lambda^{(p)}(t_1 - t_2)} \right] B^{-1}_{pq} B^{-1}_{rs}, \end{aligned} \quad (50)$$

for $t_1 > t_2$ and the stationary variance is therefore

$$V[X_i] = - \sum_{p,q,r,s} B_{ip} B_{ir} \frac{\Gamma_{qs}}{\lambda^{(p)} + \lambda^{(r)}} B^{-1}_{pq} B^{-1}_{rs}. \quad (51)$$

The solution for the input u

From Eq. 44 and 49, the input u_t satisfies

$$u_{t_2} = E[u] + \int_{t_1}^{t_2} dt' \, \mathrm{e}^{-d_U(t_2 - t')} \xi_1(t') + (u_{t_1} - E[u]) \mathrm{e}^{-dU(t_2 - t_1)} \quad (52)$$

for $t_2 > t_1$. Hence $u(t)$ depends on (is measurable with respect to) the history of $\xi_1(t)$.

Taking expectations of Eq. 52 conditional on u_{t_1} gives

$$E[u_{t_2}|u_{t_1}] = E[u] + \int_{t_1}^{t_2} dt' \, \mathrm{e}^{-d_U(t_2 - t')} E[\xi_1(t')|u_{t_1}] + (u_{t_1} - E[u]) \mathrm{e}^{-dU(t_2 - t_1)} \quad (53)$$

but $E[\xi_1(t)|u_{t_1}] = 0$ for $t > t_1$ because the value of ξ_1 at the current time is independent of the values of ξ_1 at earlier times. Hence

$$E[u_{t_2}|u_{t_1}] = E[u] + (u_{t_1} - E[u]) \mathrm{e}^{-d_U(t_2 - t_1)} \quad (54)$$

and $E[u_{t_2}|u_{t_1}]$ tends to the stationary mean $E[u]$ if $t_2 \gg t_1$, as expected. Noting that $E[u_{t_2} u_{t_1}] = E[E[u_{t_2} u_{t_1}|u_{t_1}]] = E[u_{t_1} E[u_{t_2}|u_{t_1}]]$, Eq. 54 implies

$$E[u_{t_2} u_{t_1}] - E[u]^2 = V[u] \mathrm{e}^{-d_U(t_2 - t_1)} \quad (55)$$

and the input u has an exponential autocorrelation function with an autocorrelation time of $1/d_U$, a known property of an Ornstein-Uhlenbeck process [4].

We also have (by time-reversibility of u) that

$$E[u_{t_1}|u_{t_2}] = E[u] + (u_{t_2} - E[u]) \mathrm{e}^{-d_U(t_2 - t_1)} \quad (56)$$

when $t_2 > t_1$, so that events far in the future do not affect current values of u. Taking expectations of

Eq. 52 conditional now on t_2 implies

$$u_{t_2} = E[u] + \int_{t_1}^{t_2} dt' E[\xi(t')|u_{t_2}] \mathrm{e}^{-d_U(t_2-t')} + (E[u_{t_1}|u_{t_2}] - E[u]) \mathrm{e}^{-d_U(t_2-t_1)}. \tag{57}$$

Using Eq. 56 to replace $E[u_{t_1}|u_{t_2}]$ gives

$$u_{t_2} = E[u] + \int_{t_1}^{t_2} dt' E[\xi(t')|u_{t_2}] \mathrm{e}^{-d_U(t_2-t')} + (u_{t_2} - E[u]) \mathrm{e}^{-2d_U(t_2-t_1)} \tag{58}$$

which is an equation for $E[\xi(t)|u_{t_2}]$. This equation can be solved by differentiating with respect to t_1, and we find that

$$E[\xi_1(t_1)|u_{t_2}] = 2d_U(u_{t_2} - E[u]) \mathrm{e}^{-d_U(t_2-t_1)} \tag{59}$$

which is non-zero because u_{t_2} depends on the value ξ_1 takes at time $t_1 < t_2$.

Modeling gene expression with a fluctuating transcriptional input: no feedback

To illustrate the Langevin method, we will show that the method recovers our exact results for the two-stage model of gene expression without feedback (in the case where $u(t)$ is an OU process). The model has a fluctuating rate of transcription $u(t)$ and the signal of interest, $s(t)$, is the current value of u. To the differential equation for u, Eq. 44, we must add a differential equation for mRNA and one for protein:

$$\begin{aligned} \dot{M} &= u_t - d_M M_t + \xi_2(t) \\ \dot{Z} &= v M_t - d_Z Z_t + \xi_3(t), \end{aligned} \tag{60}$$

with the ξ_i obeying the diffusion matrix

$$\Gamma = \begin{pmatrix} 2d_U V[u] & 0 & 0 \\ 0 & 2E[u] & 0 \\ 0 & 0 & 2E[u]\frac{v}{d_M} \end{pmatrix} \tag{61}$$

because $E[u] = d_M E[M]$ from Eq. 60.

Notice that

$$E[Z_t|u^{\mathcal{H}_t}] = E[Z_t|\xi_1^{\mathcal{H}_t}], \tag{62}$$

since we have the conditional independence $Z_t \perp\!\!\!\perp u^{\mathcal{H}_t}|\xi_1^{\mathcal{H}_t}$, by Eq. 49.

Determining the variance of Z

Calculating the eigenvectors and eigenvalues of the Jacobian for Eqs. 44 and 60, Eq. 51 then gives immediately that

$$V[Z] = E[Z] + \frac{d_Z}{d_M + d_Z} \frac{E[Z]^2}{E[M]} + \frac{(d_M + d_U + d_Z) v^2 V[u]}{d_M d_Z (d_M + d_U)(d_M + d_Z)(d_U + d_Z)}, \tag{63}$$

with

$$E[M] = \tfrac{E[u]}{d_M} \quad ; \quad E[Z] = \tfrac{E[u] v}{d_M d_Z}. \tag{64}$$

Conditioning Z_t on the history of u

The conditional expectation $E[Z_t|u^{\mathcal{H}_t}]$ is key to finding the fidelty errors when the signal of interest is u_t (see Eqs. 69 and 62 and 76). From Eq. 49, we find that at steady-state

$$Z_t = E[Z] + \int_0^t ds \left[v f_1(s,t)\xi_1(s) + v f_2(s,t)\xi_2(s) + \mathrm{e}^{d_Z(s-t)}\xi_3(s) \right], \tag{65}$$

where

$$f_1(s,t) = \frac{\mathrm{e}^{d_M(s-t)}}{(d_M - d_U)(d_M - d_Z)} - \frac{\mathrm{e}^{d_U(s-t)}}{(d_M - d_U)(d_U - d_Z)} + \frac{\mathrm{e}^{d_Z(s-t)}}{(d_M - d_Z)(d_U - d_Z)}, \tag{66}$$

and

$$f_2(s,t) = \frac{\mathrm{e}^{d_M(s-t)} - \mathrm{e}^{d_Z(s-t)}}{d_Z - d_M}. \tag{67}$$

To find $E[Z_t|u^{\mathcal{H}_t}]$, we can condition on the history of $\xi_1(t)$ by Eq. 62, giving

$$E[Z_t|u^{\mathcal{H}_t}] = E[Z] + v \int_0^t ds\, f_1(s,t)\xi_1(s) \tag{68}$$

because $E[\xi_{2,s}|\xi_1^{\mathcal{H}_t}] = E[\xi_{3,s}|\xi_1^{\mathcal{H}_t}] = 0$ for all $s < t$.

Determining the variance of the transformed signal

When the signal of interest is u_t, $E[Z_t|u_t]$ is the perfect representation of the signal by the output. To find $E[Z_t|u_t]$, we use the relation:

$$E[Z_t|u_t] = E\Big[E[Z_t|u^{\mathcal{H}_t}]\Big|u_t\Big]. \tag{69}$$

From Eq. 68, we therefore have

$$E[Z_t|u_t] = E[Z] + \int_0^t ds\, f_1(s,t) v E[\xi_1(s)|u_t], \tag{70}$$

or

$$E[Z_t|u_t] = E[Z] + \int_0^t ds\, f_1(s,t) v 2 d_U (u_t - E[u]) \mathrm{e}^{d_U(s-t)}, \tag{71}$$

using Eq. 59. Carrying out this integral, and taking the steady-state limit of $t \to \infty$, we find that

$$E[Z_t|u_t] = E[Z] + \frac{v(u_t - E[u])}{(d_M + d_U)(d_U + d_Z)}. \tag{72}$$

The variance of this conditional expectation is

$$V\Big[E[Z_t|u_t]\Big] = E\Big[E[Z_t|u_t]^2\Big] - \Big(E[E[Z_t|u_t]]\Big)^2, \tag{73}$$

and so from Eq. 72

$$V\Big[E[Z_t|u_t]\Big] = E[Z]^2 + \frac{V[u]v^2}{(d_M+d_U)^2(d_U+d_Z)^2} - E[Z]^2$$
$$= \frac{V[u]v^2}{(d_M+d_U)^2(d_U+d_Z)^2}. \quad (74)$$

Using $E[Z] = \frac{vE[u]}{d_M d_Z}$, Eq. 74 can be re-written as

$$V\Big[E[Z_t|u_t]\Big] = E[Z]^2 \left[\frac{d_M d_Z}{(d_M+d_U)(d_U+d_Z)}\right]^2 \eta_u^2, \quad (75)$$

with $\eta_u^2 = V[u]/E[u]^2$.

Determining the magnitude of the mechanistic error

For an input u_t, the magnitude of the mechanistic error, $E[\epsilon_m^2]$, is $E[V[Z_t|u^{\mathcal{H}_t}]]$. By definition,

$$E\Big[V[Z_t|u^{\mathcal{H}_t}]\Big] = E\Big[V[Z_t|\xi_1^{\mathcal{H}_t}]\Big]$$
$$= E\Big[E[Z_t^2|\xi_1^{\mathcal{H}_t}]\Big] - E\Big[E[Z_t|\xi_1^{\mathcal{H}_t}]^2\Big]$$
$$= E[Z^2] - E\Big[E[Z_t|\xi_1^{\mathcal{H}_t}]^2\Big]. \quad (76)$$

From Eq. 68,

$$E\Big[E[Z_t|\xi_1^{\mathcal{H}_t}]^2\Big] = E[Z]^2 + v^2 \int_0^t ds \int_0^t ds'\, f_1(s,t) f_1(s',t) E[\xi_1(s)\xi_1(s')], \quad (77)$$

which, from Eq. 45, becomes

$$E\Big[E[Z_t|\xi_1^{\mathcal{H}_t}]^2\Big] = E[Z]^2 + v^2 \int_0^t ds\, f_1(s,t)^2 2d_U V[u]. \quad (78)$$

Evaluting this integral and taking the steady-state limit of $t \to \infty$, we find

$$E\Big[E[Z_t|\xi_1^{\mathcal{H}_t}]^2\Big] = E[Z]^2 + \frac{(d_M+d_U+d_Z)v^2 V[u]}{d_M d_Z (d_M+d_U)(d_M+d_Z)(d_U+d_Z)}. \quad (79)$$

Hence, from Eq. 76 and Eq. 79,

$$E\Big[V[Z_t|u^{\mathcal{H}_t}]\Big] = V[Z] - \frac{(d_M+d_U+d_Z)v^2 V[u]}{d_M d_Z (d_M+d_U)(d_M+d_Z)(d_U+d_Z)}, \quad (80)$$

and therefore we have for the magnitude of the mechanistic error

$$E[\epsilon_m^2] = E\Big[V[Z_t|u^{\mathcal{H}_t}]\Big] = E[Z] + \frac{d_Z}{d_M+d_Z}\frac{E[Z]^2}{E[M]}, \quad (81)$$

from Eq. 63.

Determining the magnitude of the dynamical error

With a signal u_t, the magnitude of the dynamical error, $E[\epsilon_d^2]$, is $E[V[E[Z_t|u^{\mathcal{H}_t}]|u_t]]$, which is the difference of Eq. 63 and the sum of Eqs. 74 and 81,

$$E[\epsilon_d^2] = \frac{(d_M + d_U + d_Z)v^2 V[u]}{d_M d_Z (d_M + d_U)(d_M + d_Z)(d_U + d_Z)} - \frac{V[u]v^2}{(d_M + d_U)^2 (d_U + d_Z)^2} \tag{82}$$

which can be re-written as

$$E[\epsilon_d^2] = E[Z]^2 \eta_u^2 \left[\frac{d_M d_U d_Z \left\{ (d_U + d_Z)^2 + d_M (d_M + 2d_U + 3d_Z) \right\}}{(d_M + d_U)^2 (d_M + d_Z)(d_U + d_Z)^2}, \right] \tag{83}$$

using $E[Z] = \frac{vE[u]}{d_M d_Z}$.

Modeling gene expression with a fluctuating transcriptional input: negative autoregulation

When we include negative autoregulation, our system becomes

$$\begin{aligned} \dot{u}_t &= -(u_t - E[u])d_U + \xi_1(t) \\ \dot{M}_t &= \frac{u_t}{1 + K_1 Z_t} - d_M M_t + \xi_2(t) \\ \dot{Z}_t &= vM_t - d_Z Z_t + \xi_3(t) \end{aligned} \tag{84}$$

with $E[\xi_i(t)] = 0$ and

$$\Gamma = \begin{pmatrix} 2d_U V[u] & 0 & 0 \\ 0 & d_M E[M] + \frac{E[u]}{1+K_1 E[Z]} & 0 \\ 0 & 0 & vE[M] + d_Z E[Z] \end{pmatrix}. \tag{85}$$

We linearize Eq. 84 around the stationary state and proceed as before using Eqs. 49 and 51. The solution for Z_t is similar in form to Eq. 65 and Eqs. 69 and 76 still hold. The algebra is more involved and although we can find explicit solutions we do not give them here.

Modeling gene expression with a fluctuating transcriptional input: positive autoregulation

With positive autoregulation, our system is

$$\begin{aligned} \dot{u}_t &= -(u_t - E[u])d_U + \xi_1(t) \\ \dot{M}_t &= \frac{wK_1 Z_t + u_t}{1 + (K_1 + K_2) Z_t} - d_M M_t + \xi_2(t) \\ \dot{Z}_t &= vM_t - d_Z Z_t + \xi_3(t) \end{aligned} \tag{86}$$

with $E[\xi_i(t)] = 0$ and

$$\Gamma = \begin{pmatrix} 2d_U V[u] & 0 & 0 \\ 0 & d_M E[M] + \frac{wK_1 E[Z] + E[u]}{1+(K_1+K_2)E[Z]} & 0 \\ 0 & 0 & vE[M] + d_Z E[Z] \end{pmatrix}. \quad (87)$$

with w being the rate of transcription from the active promoter. We linearize Eq. 86 and proceed as before.

Analysing the sensitivity of the fidelity errors to feedback strength

We gain intuition about the effects of negative and positive feedback on signaling fidelity by plotting the transcription propensities, which are proportional to Eqs. 40 and 43, against protein level, Z, for different values of u and feedback strength, K_1 (Fig. S1). As $K_1 \to \infty$ with fixed $Z > 0$, $r(Z, u)$ tends to zero for negative feedback and to the constant w for positive feedback, limits which do not depend on the value of u. For large feedback strengths, K_1, the propensity curves become indistinguishable for different values of u (except at low values of Z) for *both* the negative and positive feedback systems. Furthermore, for positive feedback, the propensity curves are closer together at the higher values of Z favored by stronger positive feedback. We thus anticipate that strong negative and strong positive feedback will result in low fidelity.

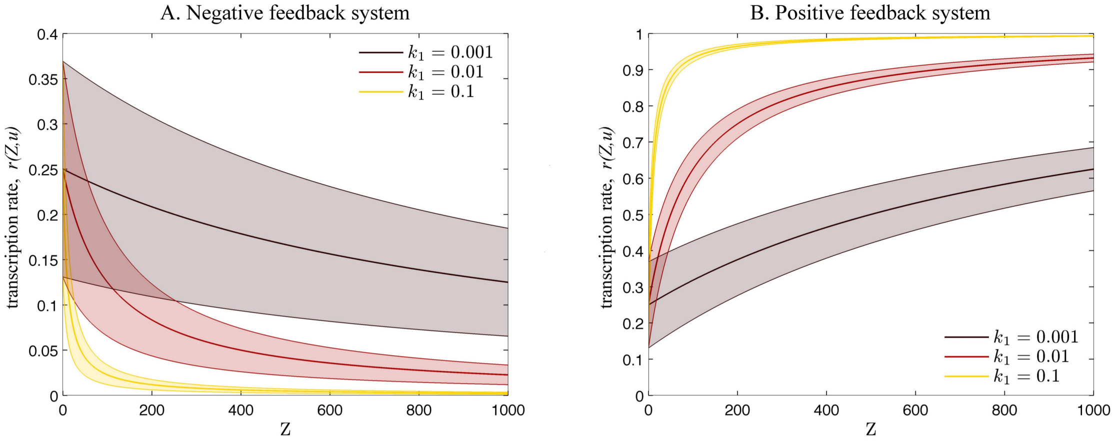

Figure S1. Transcription propensity as a function of the input value, u, and feedback strength, K_1. For different values of K_1 the transcription propensity is plotted for $u = E[u]$ (solid lines) and $u = E[u] \pm 1.96V[u]^{1/2}$ (upper and lower boundaries of the shaded areas), with $E[u]$ and $V[u]$ as in Fig. 5. (A) In the negative feedback system the transcription propensity is given by $u/\left[1 + K_1 Z\right]$. (B) In the positive feedback system the propensity is given by $\left[wK_1 Z + u\right]/\left[1 + (K_1 + K_2)Z\right]$. As K_1 increases, the propensity curves become indistinguishable for different values of u (except at low values of Z), for both the negative and positive feedback systems. For the positive feedback system we set $K_2 = 10^{-7}$ and $w = 1s^{-1}$.

For a static input and a given parametrisation of each system, the propensity curve $r(Z, u; K_1)$ determines the distribution of $Z(t)$ for the given value of the input u. As K_1 increases away from the case with no feedback ($K_1 = 0$), the propensity curves corresponding to different values of u become closer to one another (Fig. S1). We anticipate that, at least for a static input u, increasing either negative *or* positive

feedback will make it more difficult for the system to discriminate between different values of the input and signaling fidelity will deteriorate.

In the limit of a 'static' input

We want to analyze the case where fluctuations in the input $u(t)$ are sufficiently slow that the input is effectively constant over the timescales of relevance for the biochemical system. To do this, we analyse the variance components and fidelity errors from the Langevin analysis in the limit $d_u \to 0$, that is in the limit of large autocorrelation time, ${d_u}^{-1}$. We hold other parameters constant, including the variance of $u(t)$.

As we expect, both dynamical errors, $E[{\varepsilon_d}^2(t)]$ and $E[{\tilde{\varepsilon}_d}^2(t)]$, converge to zero in this limit. The fluctuations in $u(t)$ are too slow to give rise to any dynamical error. The partial derivatives with respect to feedback strength, K_1, of the (limiting) variance of the transformed signal, $V\{E[Z(t)|u(t)]\}$, are given by

$$-\frac{4v^3E[u]V[u]}{d_M d_Z (d_M d_Z K_1^{-1} + 4vE[u])^2 K_1^2} \tag{88}$$

for negative feedback and by

$$-\frac{v^2(-2d_M d_Z(1-2E[u])v + 2K_1 v^2)V[u]}{(d_M^2 d_Z^2 - 2d_M d_Z(K_1 - 2(K_1+K_2)E[u])v + K_1^2 v^2)^2}, \tag{89}$$

for positive feedback, where we have rescaled time so that w is equal to one by definition for all parametrisations of the system.

The 'signal' variance is therefore decreasing in K_1 for all feedback strengths for negative autoregulation, and decreasing for all feedback strengths $K_1 > \frac{d_M d_Z}{v}(1-2E[u])$ for positive autoregulation. In the latter case, non-monotonic behavior of the 'signal' variance occurs if $E[u] < \frac{1}{2}$ and K_1 can be made sufficiently small without violating the condition for positive feedback, $u(t) < \frac{K_1}{K_1+K_2}$. The effect on fidelity of the changes in the variance of the transformed signal depends on how the mechanistic variance changes for each type of feedback (and on the initial levels of the variance components).

The sensitivities to feedback strength of the mechanistic variance component and of the (relative) fidelity errors are complicated. We determine the signs of the relevant partial derivatives for the biophysically plausible parameter space given in Table S1, excluding the parameter d_u, and using the sampling procedure described immediately below.

	d_M	d_Z	d_U	$E[u(t)]$	$V[u(t)]^{\frac{1}{2}}$	v	K_2
Minimum	6.4×10^{-4}	5×10^{-5}	1×10^{-6}	1×10^{-3}	$E[u(t)]/[3.29 \times 10]$	4×10^{-3}	1×10^{-7}
Maximum	1.1×10^{-2}	4×10^{-4}	1×10^{-3}	5×10^{-1}	$E[u(t)]/3.29$	5×10^{-2}	1×10^{-5}

Table S1. The biophysically plausible parameter space. The minimum and maximum of the interval of values allowed for each parameter are reported. The parameters are variation free, that is, the parameter space is given by the Cartesian product of these intervals. The units of all parameters are s^{-1} (and per molecule where relevant). The maximum standard deviation of $u(t)$ is set so that, when $u(t)$ is the Gaussian Ornstein-Uhlenbeck process, the probability of $u(t) \leq 0$ is bounded above by 0.001 (no matter what value of $V[u(t)]^{\frac{1}{2}}$ is sampled). For the positive feedback system, we set $w = 1s^{-1}$ and the minimum K_1 considered is set so that $u(t) < w\frac{K_1}{K_1+K_2}$ with a probability that is bounded below by 0.999. For the negative feedback system, $K_1 \in [10^{-3}, 10]$.

As expected, we find that the mechanistic variance is always decreasing in feedback strength, K_1, for negative feedback [8] and always increasing for positive feedback (500 models sampled for both systems). For negative feedback, the (relative) fidelity error $E[\tilde{\varepsilon}_f(t)^2]$ (equivalently, the mechanistic error) always increases as K_1 is increased. The mechanistic variance does not decrease sufficiently strongly to outweigh the effect of the decreased 'signal' variance. For positive feedback, the relative fidelity error is again typically increasing in the feedback strength for all values of K_1 in the parameter space (88% of models sampled), despite the non-monotonic behavior of the 'signal' variance (observed for 97% of models sampled). The reduction in fidelity error, $E[\tilde{\varepsilon}_m(t)^2]$, from using a value of K_1 greater than the minimal one allowed was less than 0.001 for all models sampled.

With a fluctuating transcriptional input

The analytical sensitivities for the fidelity error magnitudes are again complicated. We therefore determine their signs for the biophysically plausible parameter space given in Table S1. The parameter space is the same for the negative and positive feedback systems, except for the absence of the parameter K_2 in the case of negative feedback. We choose the allowed ranges for each parameter using values typical for *E. coli* and relying on experimental measurements reported in the literature wherever possible (see [8]). We randomly and uniformly sample models from the parameter space (1000 negative feedback and 500 positive feedback models) and ask how the dynamical, mechanistic and fidelity errors change as the feedback strength K_1 is varied. As we sample, the parameter space governing fluctuations of $u(t)$ is also explored. Specifically, we determine the signs of the analytical partial derivatives of the magnitudes of the (relative) dynamical, mechanistic and fidelity errors with respect to feedback strength, K_1. We do this for values of K_1 in a predetermined range (i.e., without sampling values of K_1, see Table S1), for each parametrisation of the system that is sampled, by maximizing or minimizing the relevant partial derivative with respect to K_1. We also determine, for each such system, the value of K_1 that minimizes the (relative) fidelity error. The results are discussed in the main text.

The effect on fidelity of varying feedback strength with the gain held constant

In our feedback models, the faithfully transformed signal is a linear function of $u(t)$: $E[Z(t)|u(t)] = c + gu(t)$, for constants c and g. We define g to be the gain of the network for the signal of interest, $u(t)$. The variance of the transformed signal or 'dynamic range', $V\{E[Z(t)|u(t)]\}$, is equal to $g^2V[u(t)]$. Varying K_1 alone (with $V[u(t)]$ held constant), the gain and hence the variance of the transformed signal also vary.

We investigated the effect of varying K_1 while simultaneously altering the translation rate (v) in order to hold the gain constant. Since we do not in general have v as an explicit function of K_1, we can no longer compute the sensitivities of the fidelity errors to K_1. Instead, starting with a randomly sampled parameter vector, we numerically minimise the (relative) fidelity error, $E[\tilde{\varepsilon}_f(t)^2]$, with respect to K_1. At each step of the minimisation procedure, a new K_1 is chosen, together with a value of v which results in the same gain (as that for the initially sampled parameter vector). We then compare the fidelity errors corresponding to the *optimal* K_1 and to the initially sampled parameter vector.

For the negative feedback models (both with static and dynamic inputs), we sampled 500 parameter vectors at random from the biophysically plausible parameter space in Table S1, and optimised K_1 over the same range of values considered throughout ($K_1 \in [10^{-3}, 10]$). In the static case, we find almost always that the absolute mechanistic error increases and the fidelity therefore decreases if the feedback strength, K_1, is increased ($> 99.5\%$ of models sampled): hence the optimal K_1 is the minimum allowed value. When the input is dynamic, we find the (relative and absolute) dynamical error decreases with increased K_1 while the (relative and absolute) mechanistic error increases (and vice versa when K_1 is decreased compared to its initial value). However, in the dynamic case there is now usually an optimal feedback strength, K_1, below and above which fidelity deteriorates ($> 98\%$ of models sampled).

In our hands, the analogous procedure for the positive feedback model did not result in a convergent numerical optimisation procedure, perhaps owing to the multiple solutions that exist for translation rates to achieve constant gain.

Quantifying fidelity errors using a numerical, Monte Carlo method

We can evaluate the magnitudes of the fidelity errors (Eq. 6 main text) for any signaling network and choice of the signal of interest, $s(t)$, provided that we can simulate the input process, $u(t)$ (and given the necessary computational resources). Using an extension of the 'Gillespie' simulation algorithm that allows propensities to vary stochastically between reaction times [9], we include in the simulated reaction network a 'conjugate reporter' $Z_2(t)$ that is independent of and identically distributed to the output $Z(t)$, conditional upon the input history $u^{\mathcal{H}_t}$. This requires *in silico* duplication of the network 'downstream' of the input $u(t)$. The average squared deviation of the 2 reporters then identifies the mechanistic error [1], since

$$\frac{1}{2}E\{[Z(t) - Z_2(t)]^2\} = E\{V[Z(t)|u^{\mathcal{H}_t}\} = E[\varepsilon_m(t)^2].$$

The mechanistic error can be estimated by taking the sample average of $[Z(t)-Z_2(t)]^2$ over different times t using a single simulated realisation of the system (in the ergodic case) or by independently generating multiple realisations of the system (including the input u) and taking the ensemble average at time t. We evaluate the dynamical error indirectly, by obtaining a simulated sample from the distribution of the signal-output pair, $[s(t), Z(t)]$, and then estimating the conditional means $E[Z(t)|s(t)]$. When $s(t)$ has a small number of possible states, the conditional means can be estimated by the average of the outputs corresponding to each input state. Otherwise, a flexible 'curve-fitting' or nonparametric regression technique is used to estimate the function $E[Z(t)|s(t)]$. Using the known distribution of $s(t)$, possibly the empirical one from the simulation output, we can then estimate the variance of $E[Z(t)|s(t)]$. Finally, we evaluate the dynamical error using $E[\varepsilon_d(t)^2] = V[Z(t)] - V\{E[Z(t)|s(t)]\} - E[\varepsilon_m(t)^2]$, by Eq.6.

Figure S2 illustrates the effect of positive feedback on signaling fidelity when the input process, $u(t)$, switches between two states (u_h and u_l) according to a two-state Markov Chain (or 'random telegraph process') and $s(t) = u(t)$. It is the analogue of Fig. 4, which is for negative autoregulation. As for negative feedback, increasing positive feedback strength reduces dynamical error but increases mechanistic error, and overall fidelity deteriorates. For both figures, the protein level of each conjugate reporter was sampled at the times t_i ($i = 1, ..., N$), with the frequency of sampling set as $t_{i+1} - t_i = 10d_u^{-1}$, where d_u^{-1} is the autocorrelation time of $u(t)$. The sample population was divided into two parts, S_l and S_h, based on the

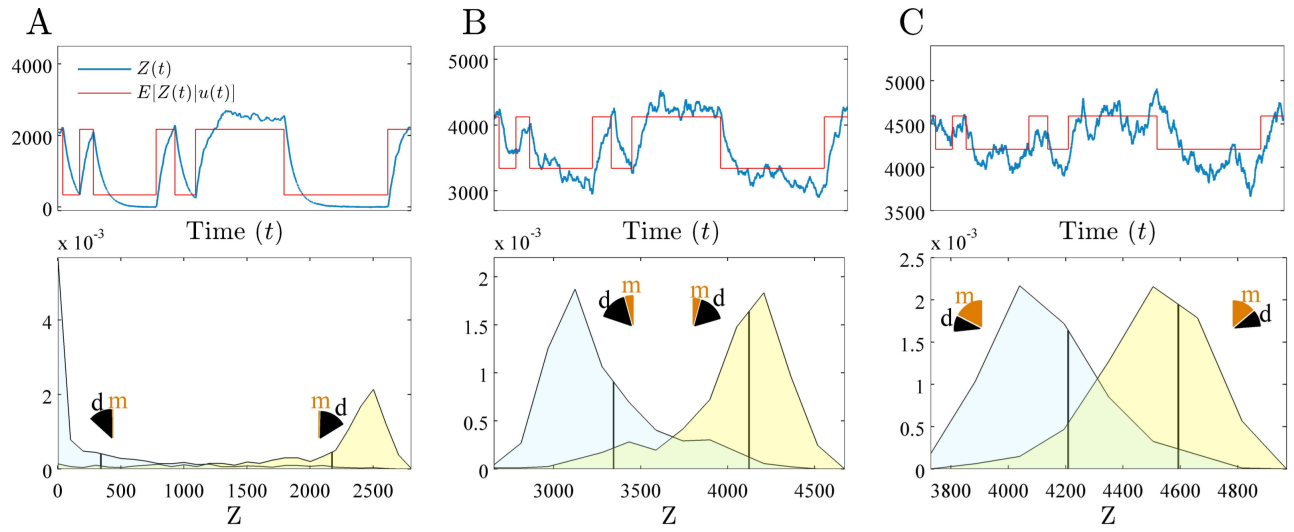

Figure S2. Increasing positive feedback strength harms signaling fidelity. We consider a 2-stage model of gene expression with the signal of interest, $s(t)$, being the value of the current input, $u(t)$, which is proportional to the level of a transcriptional activator. We simulate $u(t)$ as in Fig. 1A. Upper row compares the time course of the protein output (blue) to the faithfully transformed signal (red), $E[Z(t)|u(t)]$. Lower row shows the distributions for the output, Z, that correspond to each of the two possible values of the input, u (low and high). Vertical lines indicate the means of the distributions. Pie charts show the fractions of the variance of each (conditional) distribution due to dynamical (d) and mechanistic (m) error, weighted by the probability of the input state: summing these gives the overall magnitude (variance) of the dynamical and mechanistic errors. (A) no feedback, $K_1 = 0$. (B) intermediate positive feedback, $K_1 = 5 \cdot 10^{-4}$, $K_2 = 10^{-7}$. (C) strong positive feedback, $K_1 = 10^{-3}$, $K_2 = 10^{-7}$. As the strength of feedback increases, (relative) mechanistic error increases and the underlying state of the input becomes more difficult to infer (the conditional distributions overlap more). Transcription propensities are given by $[wK_1Z(t) + u(t)] / [1 + (K_1 + K_2)Z(t)]$ with $w = 1$, and all parameters except K_1 and K_2 and are as in Fig. 4.

state of the signal $u(t)$ at the time of sampling. The two samples were used to estimate the conditional distributions shown in Figures S2 and 4 (lower rows) as well as the conditional mean $E[Z(t)|u(t) = u_.]$ and variance $V[Z(t)|u(t) = u_.]$ for the two possible values of the signal $u_. \in u_l, u_h$. To calculate the magnitude of the conditional fidelity errors we use the expression

$$V[Z(t)|u(t) = u_.] = E[\varepsilon^2{}_m(t)|u(t) = u_.] + E[\varepsilon^2{}_d(t)|u(t) = u_.]. \tag{90}$$

That is, the conditional variance of $Z(t)$ can be decomposed into two terms describing the magnitude of the conditional mechanistic and conditional dynamical error. Using a long simulation time, the first term can be found from the sample average of the squared difference between the two conjugate reporters, $|S_.|^{-1} \sum_{i \in S_.} [Z(t_i) - Z_2(t_i)]^2/2$. Then, the second term corresponds to the difference between the estimated conditional variance and the estimated conditional mechanistic error. Note that the magnitude of the total mechanistic or dynamical error is obtained by taking the average of the corresponding conditional ones using the stationary distribution of $u(t)$.

As a further illustration, we also applied our general numerical method for quantifying fidelity errors when $u(t)$ is a Gaussian Ornstein-Uhlenbeck process. We are thus able to assess the adequacy of the Langevin approximations for the negative and positive feedback systems. Using a long simulation time,

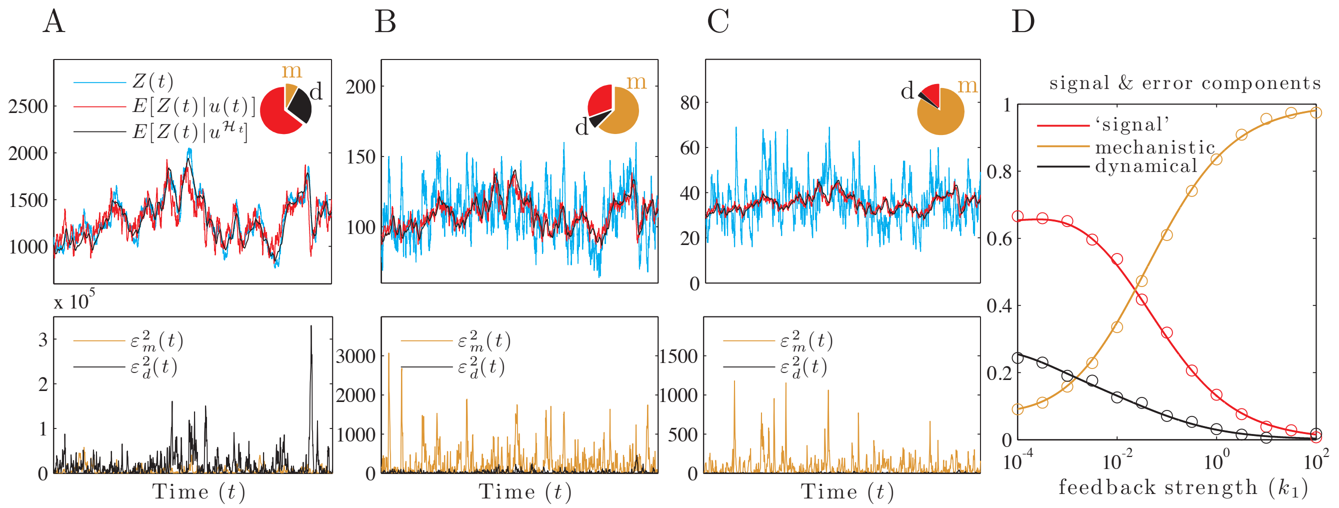

Figure S3. The deterioration in signaling fidelity when negative feedback strength increases is well described by the Langevin analysis. We consider a 2-stage model of gene expression with the signal of interest $s(t) = u(t)$, and with $u(t)$ proportional to the level of a transcriptional activator and modeled as an Ornstein-Uhlenbeck process. Upper row in panels A to C compares the time course of the protein output (blue) to the perfect signal representation, $E[Z(t)|u(t)]$ (red), and to the expected output given the input history, $E[Z(t)|u^{\mathcal{H}_t}]$ (black). Pie chart insets show the fractions of the protein variance due to the mechanistic (m) and dynamical (d) errors, and due to the transformed signal. Lower row shows time courses of the error magnitudes. (A) no feedback, $K_1 = 0$ (B) intermediate negative feedback ($K_1 = 0.1$). (C) strong negative feedback ($K_1 = 1$). (D) The magnitude of the dynamical error, $\tilde{\varepsilon}_d(t)$, and of the mechanistic error, $\tilde{\varepsilon}_m(t)$, as a function of the feedback strength, K_1 (the 'signal' component is 1 minus the magnitude of the (relative) fidelity error). Circles are for the numerical procedure and solid lines for the Langevin analysis. Transcription propensities are given by $u(t)/[1 + K_1 Z(t)]$. All parameters except K_1 are as in Fig. 5.

we estimate $V[Z(t)]$ by the sample variance of one of the conjugate reporters, and estimate $E[\varepsilon^2{}_m(t)]$ using the 2 conjugate reporters by the sample average $N^{-1}\sum_{i=1}^{N}[Z(t_i) - Z_2(t_i)]^2/2$. The estimation of the regression function $E[Z(t)|u(t)]$ was performed for the simulated sample $\{[Z(t_i), u(t_i)]; i = 1, ..., N\}$ using a cubic smoothing spline (Matlab built-in function *csaps* with default parameters). Figures S3 and S4 illustrate the close agreement between the numerical results and those obtained using the Langevin theory, for both the negative and positive feedback systems.

Combining outputs from multiple cells improves fidelity

We consider the signaling fidelity achieved by the average or aggregate output of a group of N identical cells receiving the same fluctuating input, $u(t)$. The absolute dynamical error for the average output is the same as that for each individual cell because

$$E[\bar{Z}(t)|u^{\mathcal{H}_t}] = E[Z_i(t)|u^{\mathcal{H}_t}], \tag{91}$$

where $\bar{Z}(t) = N^{-1}\sum_{i=1}^{N} Z_i(t)$ is the average output of the group, and $Z_i(t)$ is the output of the ith individual cell. Therefore, after taking conditional expectations with respect to $s(t)$ in Eq. 91, we also have that $E[\bar{Z}(t)|s(t)] = E[Z_i(t)|s(t)]$, since $s(t)$ is known when the history of u at time t is known

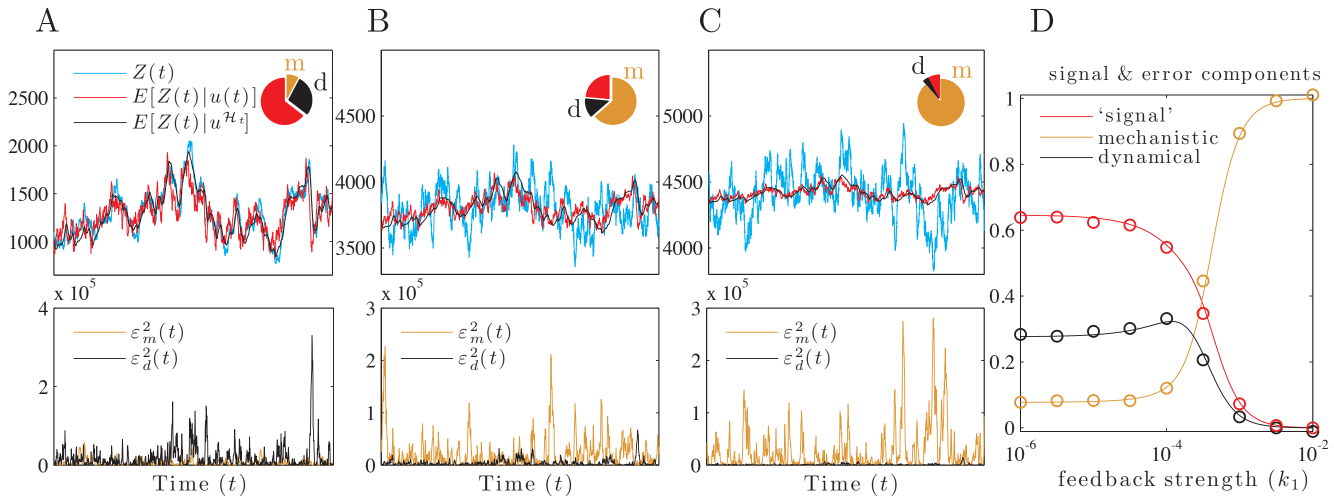

Figure S4. The deterioration in signaling fidelity when positive feedback strength increases is well described by the Langevin analysis. We consider a 2-stage model of gene expression with the signal of interest $s(t) = u(t)$, and with $u(t)$ proportional to the level of a transcriptional activator and modeled as an Ornstein-Uhlenbeck process. Upper row in panels A to C compares the time course of the protein output (blue) to the perfect signal representation, $E[Z(t)|u(t)]$ (red), and to the expected output given the input history, $E[Z(t)|u^{\mathcal{H}_t}]$ (black). Pie chart insets show the fractions of the protein variance due to the mechanistic (m) and dynamical (d) errors, and due to the transformed signal. Lower row shows time courses of the error magnitudes. (A) no feedback, $K_1 = 0$. (B) intermediate positive feedback ($K_1 = 5 \cdot 10^{-4}$). (C) strong positive feedback ($K_1 = 10^{-3}$). (D) The magnitude of the dynamical error, $\tilde{\varepsilon}_d(t)$, and of the mechanistic error, $\tilde{\varepsilon}_m(t)$, as a function of the feedback strength, K_1 (the 'signal' component is 1 minus the magnitude of the (relative) fidelity error). Circles are for the numerical procedure and solid lines for the Langevin analysis. Transcription propensities are given by $[wK_1Z(t) + u(t)]\,/\,[1 + (K_1 + K_2)Z(t)]$, with $K_2 = 10^{-7}$ and $w = 1$ throughout the figure. All parameters except K_1, K_2 are as in Fig. 5.

($\sigma[s(t)] \subseteq u^{\mathcal{H}_t}$). The dynamical error is therefore unchanged and equal to $\varepsilon_{d,i}(t)$, that for an individual cell.

When the cells are also independent (given $u^{\mathcal{H}_t}$), the fidelity for both the average output of the group, $\bar{Z}(t)$, and for the aggregate output, $\sum_{i=1}^{N} Z_i(t)$, becomes

$$\text{fidelity}_{\text{group}} = \frac{V\{E[Z_i(t)|s(t)]\}}{E[\varepsilon_{d,i}^2(t)] + N^{-1}E[\varepsilon_{m,i}^2(t)]}, \tag{92}$$

where $\varepsilon_{m,i}(t)$ is the mechanistic error for an individual cell and we have again used $E[\bar{Z}(t)|s(t)] = E[Z_i(t)|s(t)]$ to obtain the numerator. Note that the (absolute) mechanistic error for the average output of the group has magnitude equal to $E\{V[\bar{Z}(t)|u^{\mathcal{H}_t}]\} = N^{-1}E[\varepsilon_{m,i}^2(t)]$. Our theory thus predicts that fidelity must be higher for the group of independent, identical cells, by comparing Eq.'s 7 and 92.

Designing dynamic synthetic networks

Suppose the target response for a synthetic network is given by $r(t)$, which is a function of the input trajectory, $u^{\mathcal{H}_t}$. We can think of the target as a response to some particular feature of the input history, the signal of interest $s(t)$, and write $r(t) = R(s(t))$. Sometimes, network responses differing only by

scaling constants will have very different desirability in a synthetic setting. For example, when $R(s(t))$ gives the target number of molecules for secretion by the cell. It is then appropriate to use the magnitudes of the absolute fidelity errors rather than those of the relative errors. Eq. 5 is readily extended to give the following decomposition of the discrepancy between the actual and target network responses:

$$Z(t) - r(t) = \{E[Z(t)|s(t)] - r(t)\} + \varepsilon_d(t) + \varepsilon_m(t), \tag{93}$$

for all $t \geq 0$, where the definitions and interpretations of the two errors $\varepsilon_d(t)$ and $\varepsilon_m(t)$ are unchanged. There is now an additional error term, $\varepsilon_r(t) := \{E[Z(t)|s(t)] - r(t)\}$, arising from the possible discrepancy between the conditional mean and the ideal response $R(s(t))$. The magnitude of the fidelity error becomes the objective to be minimised by the synthetic design and decomposes as

$$E\{[Z(t) - r(t)]^2\} = E[\varepsilon_r(t)]^2 + E[\varepsilon_d(t)^2] + E[\varepsilon_m(t)^2]. \tag{94}$$

References

1. Bowsher CG, Swain PS (2012) Identifying information flow and sources of variation in biochemical networks. *Proc Natl Acad Sci USA* 109:E1320–E1328.

2. Hilfinger A, Paulsson J (2011) Separating intrinsic from extrinsic fluctuations in dynamic biological systems. *Proc Natl Acad Sci USA* 108:12167–72.

3. Ackers G, Johnson A, Shea M (1982) Quantitative model for gene regulation by lambda phage repressor. *Proc Natl Acad Sci USA* 79:1129–1133.

4. van Kampen NG (1992) *Stochastic Processes in Physics and Chemistry* (Elsevier).

5. Gillespie DT (2000) The chemical Langevin equation. *The Journal of Chemical Physics* 113:297.

6. Elf J, Ehrenberg M (2003) Fast evaluation of fluctuations in biochemical networks with the linear noise approximation. *Genome Research* 13:2475–2484.

7. Swain PS (2004) Efficient attenuation of stochasticity in gene expression through post-transcriptional control. *J Mol Biol* 344:965–76.

8. Voliotis M, Bowsher CG (2012) The magnitude and colour of noise in genetic negative feedback systems. *Nucleic Acids Res* 40:7084–95.

9. Shahrezaei V, Ollivier JF, Swain PS (2008) Colored extrinsic fluctuations and stochastic gene expression. *Mol Syst Biol* 4:196.
